# Supplementary material for: Development and Validation of a Visualized Posture Risk Assessment Questionnaire for Low Back Pain in Daily Activities: A Study in Taiwan
Source: Healthcare (Basel). 2024 Nov 14;12(22):2274. doi: 10.3390/healthcare12222274 (PMC11593931; doi:10.3390/healthcare12222274)
Supplement: Supplementary file 1 [file healthcare-12-02274-s001.zip › healthcare-3279036-supplementary/Q_TC_ENG/Q_ENG_20241003.pdf]

Daily Activities and Posture Risk Assessment Questionnaire for Low Back Pain

Please reflect on the past two weeks of your daily activities and indicate how frequently you performed each posture by selecting the appropriate option. Your responses will help us assess your posture habits and provide tailored professional advice.

Frequency in the Past Two Weeks<br>Please indicate your response<br>by checking (✔) the appropriate box.	Usually<br>(≥10 times)	Frequently<br>(5-9 times)	Occasionally<br>(1-4 times)	Never<br>(0 times)
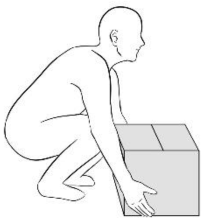<br>Lifting heavy objects<br>(Lifting with knees bent and back straight while squatting)				
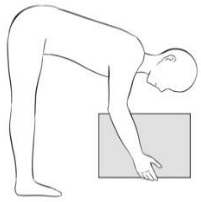<br>Lifting heavy objects<br>(Lifting with knees straight and back bent while leaning forward)				
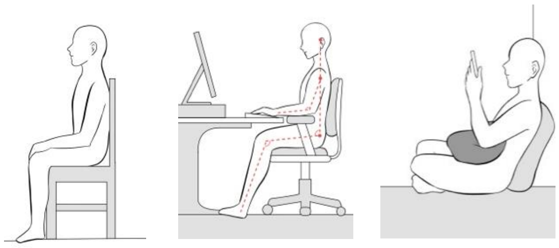<br>Sitting<br>(Sitting upright with your back resting fully against the chair)				
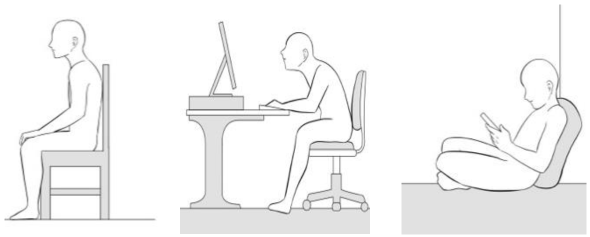<br>Sitting<br>(Slouching posture with your back hunched and not resting fully against the chair)				

Frequency in the Past Two Weeks<br>Please indicate your response<br>by checking (✔) the appropriate box.	Usually<br>(≥10 times)	Frequently<br>(5-9 times)	Occasionally<br>(1-4 times)	Never<br>(0 times)
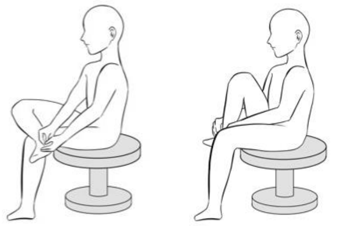<br>Putting on shoes<br>(Putting on shoes with knees bent<br>and back straight)				
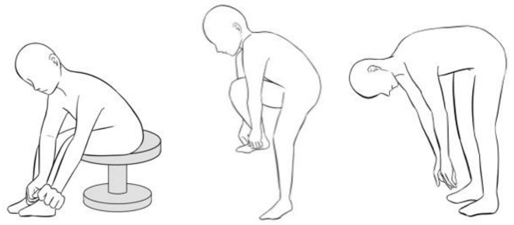<br>Putting on shoes<br>(Putting on shoes while sitting, lifting your feet, and<br>keeping your waist straight)				
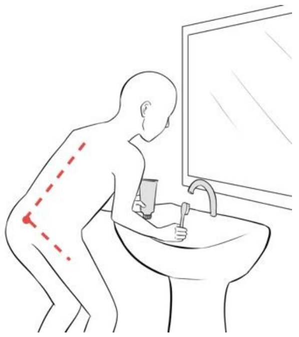<br>Face washing and tooth brushing<br>(Washing face or brushing teeth with<br>slightly bent knees and a straight back)				
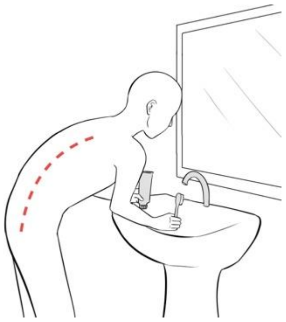<br>Face washing and tooth brushing<br>(Washing face or brushing teeth with knees straight<br>and leaning forward with a bent back)				

Frequency in the Past Two Weeks<br>Please indicate your response<br>by checking (✔) the appropriate box.	Usually<br>(≥10 times)	Frequently<br>(5-9 times)	Occasionally<br>(1-4 times)	Never<br>(0 times)
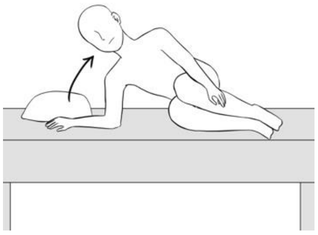<br>Getting out of bed<br>(Rolling to your side first and<br>pushing yourself up with your hand)				
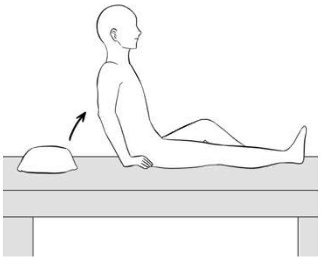<br>Getting out of bed<br>(Sitting up directly by bending at the waist)				
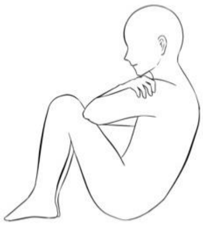<br>Doing sit-ups<br>(Performing a sit-up by lifting<br>the entire back off the bed)				
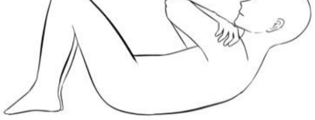<br>Doing sit-ups<br>(Performing a sit-up by lifting<br>only the shoulder blades off the bed)				
