# Supplementary material for: Development and Validation of a Visualized Posture Risk Assessment Questionnaire for Low Back Pain in Daily Activities: A Study in Taiwan
Source: Healthcare (Basel). 2024 Nov 14;12(22):2274. doi: 10.3390/healthcare12222274 (PMC11593931; doi:10.3390/healthcare12222274)
Supplement: Supplementary file 1 [file healthcare-12-02274-s001.zip › healthcare-3279036-supplementary/Q_TC_ENG/Q_TC_20241003.pdf]

# 下背痛之日常活動與姿勢風險評估問卷

為了瞭解您日常生活中經常使用的姿勢與動作，我們希望您回想過去兩週的日常生活，並根據您的記憶填寫以下問卷，回憶您在日常生活中各種姿勢或動作出現的頻率，這將幫助我們更完整的瞭解您的日常生活姿勢，並提供專業建議。

| 過去兩週的頻率<br>請您已打勾（V）的方式填寫 | 習慣這麼做<br>(10次以上) | 常常這麼做<br>(5-9次之間) | 偶爾這麼做<br>(1-4次之間) | 從不這麼做<br>(0次) |
|---|---|---|---|---|
| 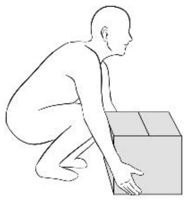<br>搬重物時，腳蹲下，身體去搬<br>(膝蓋彎曲，身體直立蹲下) | | | | |
| 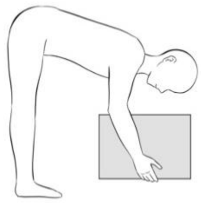<br>搬重物時，直接彎腰，身體去搬<br>(膝蓋打直，直接彎腰) | | | | |
| 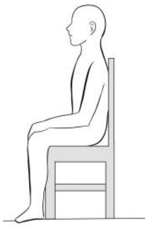 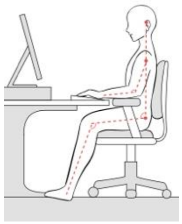 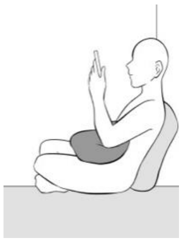<br>坐姿<br>(身體坐正，腰倚靠著椅背) | | | | |
| 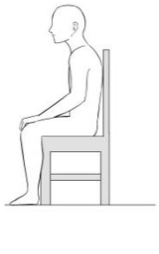 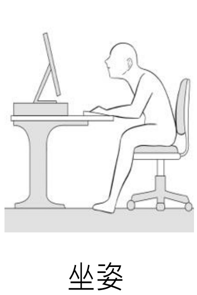 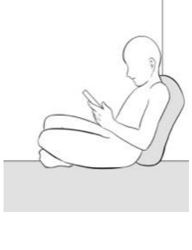<br>坐姿<br>(慵懶坐姿，駝背及背部沒有平均以靠椅背) | | | | |

| 過去兩週的頻率<br>請您已打勾 ( V ) 的方式填寫 | 習慣這麼做<br>(10次以上) | 常常這麼做<br>(5-9次之間) | 偶爾這麼做<br>(1-4次之間) | 從不這麼做<br>(0次) |
|---|---|---|---|---|
| 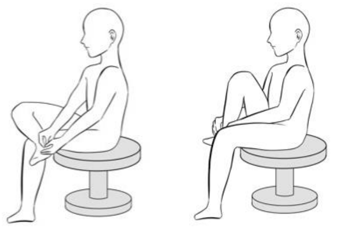<br>用這樣的姿勢穿鞋<br>(坐姿，把腳抬高，腰背挺直，或蹺腳腰背挺直) | | | | |
| 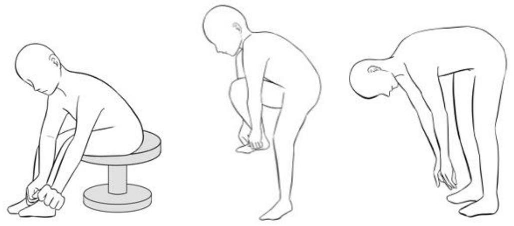<br>用這樣的姿勢穿鞋<br>(單膝或雙膝的膝蓋打直，直接彎腰穿鞋) | | | | |
| 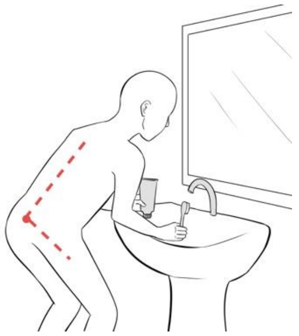<br>用這樣的姿勢洗臉刷牙<br>(膝蓋微彎曲，身體直立向前) | | | | |
| 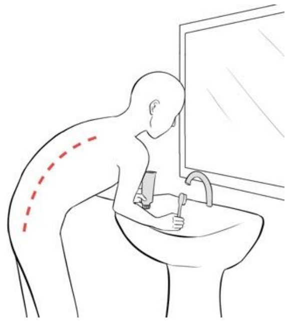<br>用這樣的姿勢洗臉刷牙<br>(膝蓋伸直，身體彎腰向前) | | | | |

| 過去兩週的頻率<br>請您已打勾（V）的方式填寫 | 習慣這麼做<br>(10次以上) | 常常這麼做<br>(5-9次之間) | 偶爾這麼做<br>(1-4次之間) | 從不這麼做<br>(0次) |
|---|---|---|---|---|
| 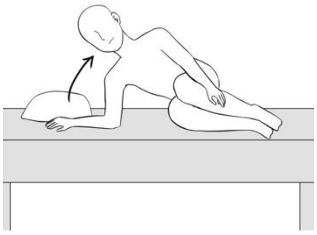<br>從床上坐起來的方式<br>(先側躺，再用手撐起身體) | | | | |
| 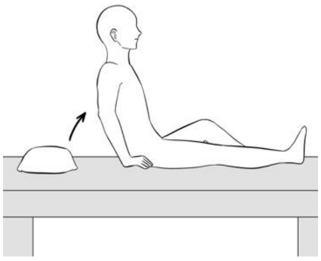<br>從床上坐起來的方式<br>(直接彎腰坐起) | | | | |
| 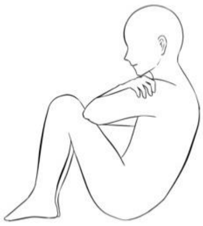<br>仰臥起坐<br>(可以做到整個背部抬離床面) | | | | |
| 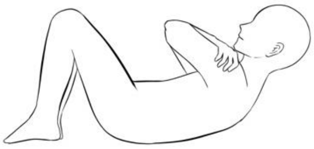<br>仰臥起坐<br>(只有肩胛骨以上部位可以抬離床面) | | | | |
