# Supplementary figures and images for: Development and Validation of a Visualized Posture Risk Assessment Questionnaire for Low Back Pain in Daily Activities: A Study in Taiwan
Source: Healthcare (Basel). 2024 Nov 14;12(22):2274. doi: 10.3390/healthcare12222274 (PMC11593931; doi:10.3390/healthcare12222274)

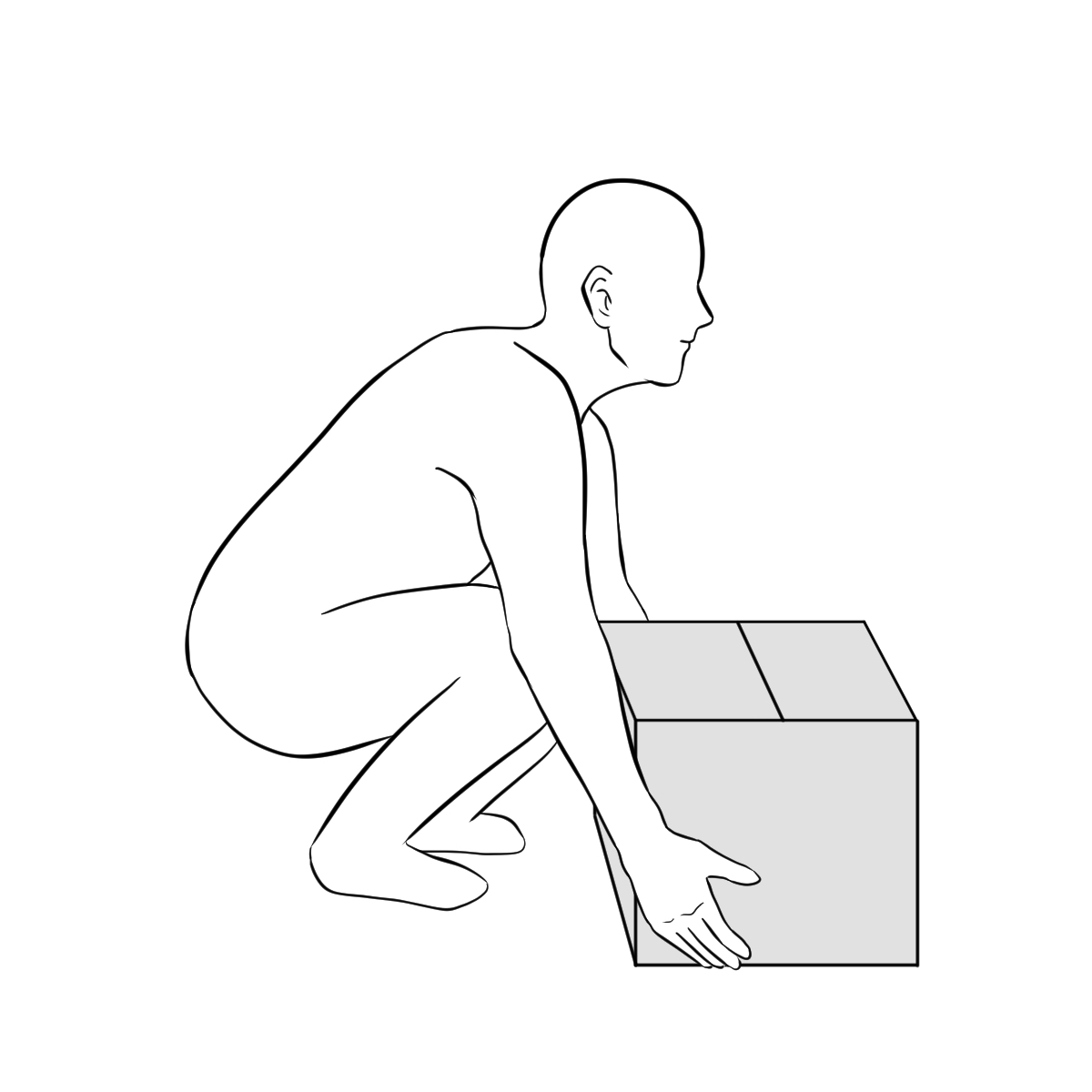

Supplement: Supplementary file 1 [file healthcare-12-02274-s001.zip › healthcare-3279036-supplementary/healthcare20241108_Postures/1-1.jpg]

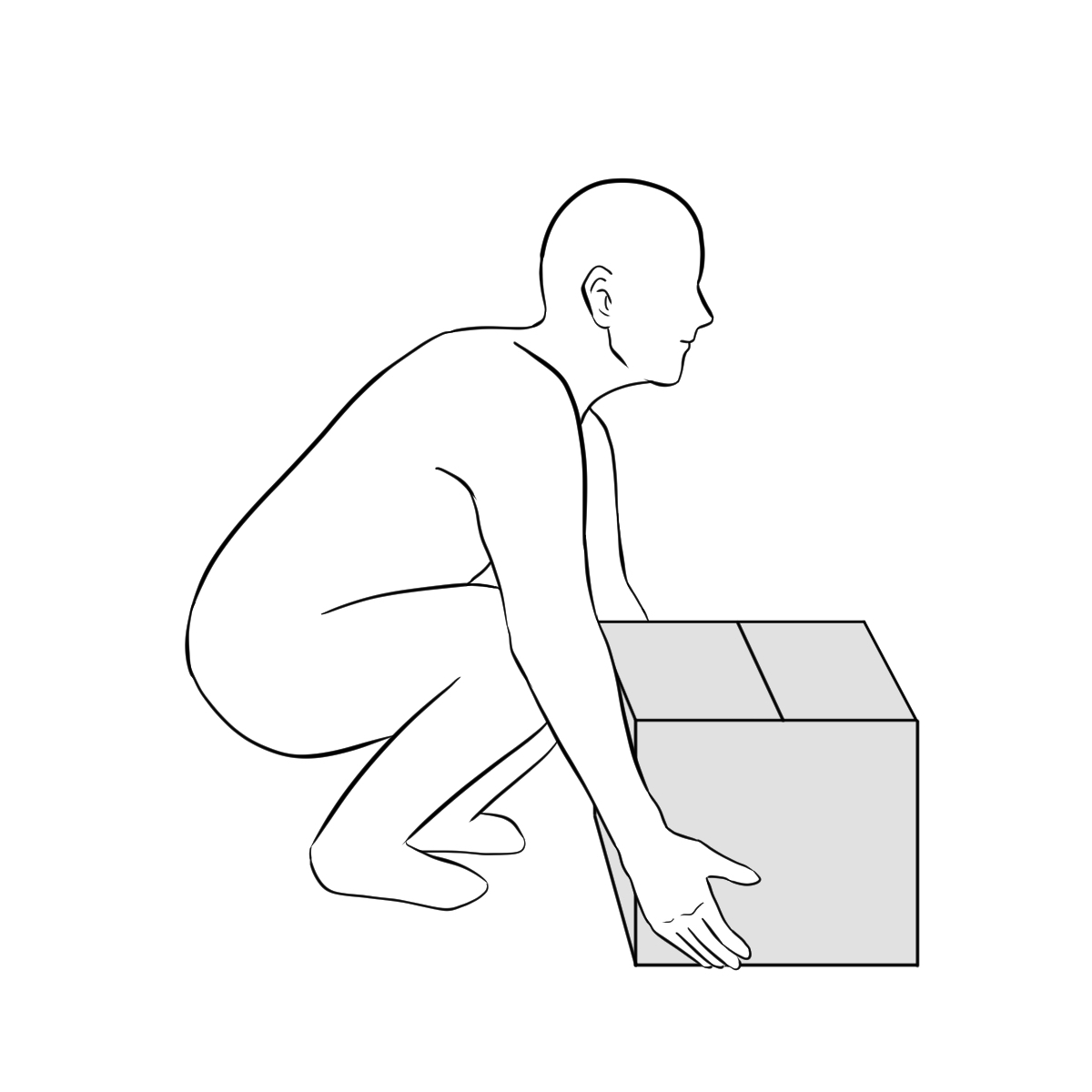

Supplement: Supplementary file 1 [file healthcare-12-02274-s001.zip › healthcare-3279036-supplementary/healthcare20241108_Postures/1-1_TIFF.tif]

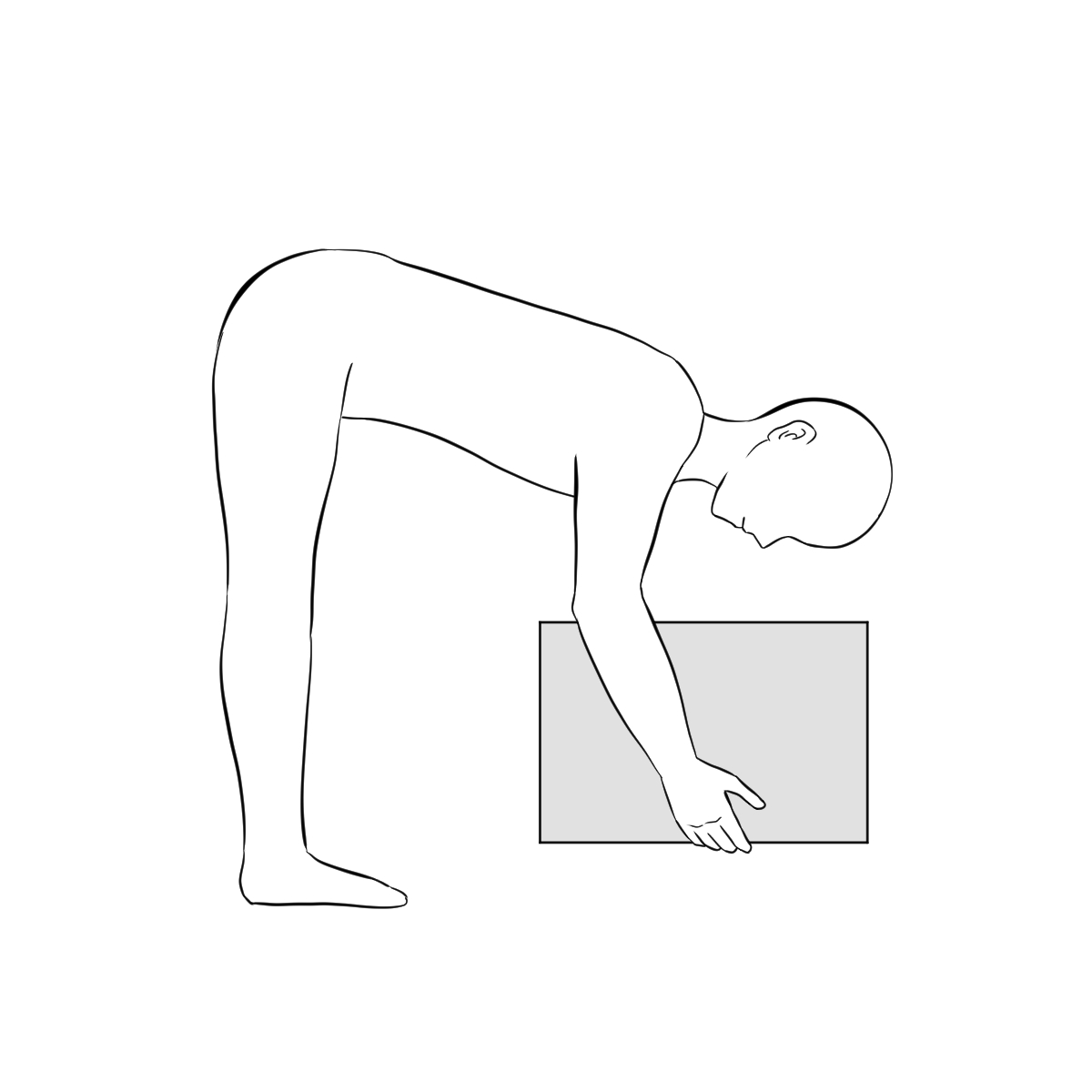

Supplement: Supplementary file 1 [file healthcare-12-02274-s001.zip › healthcare-3279036-supplementary/healthcare20241108_Postures/1-2.jpg]

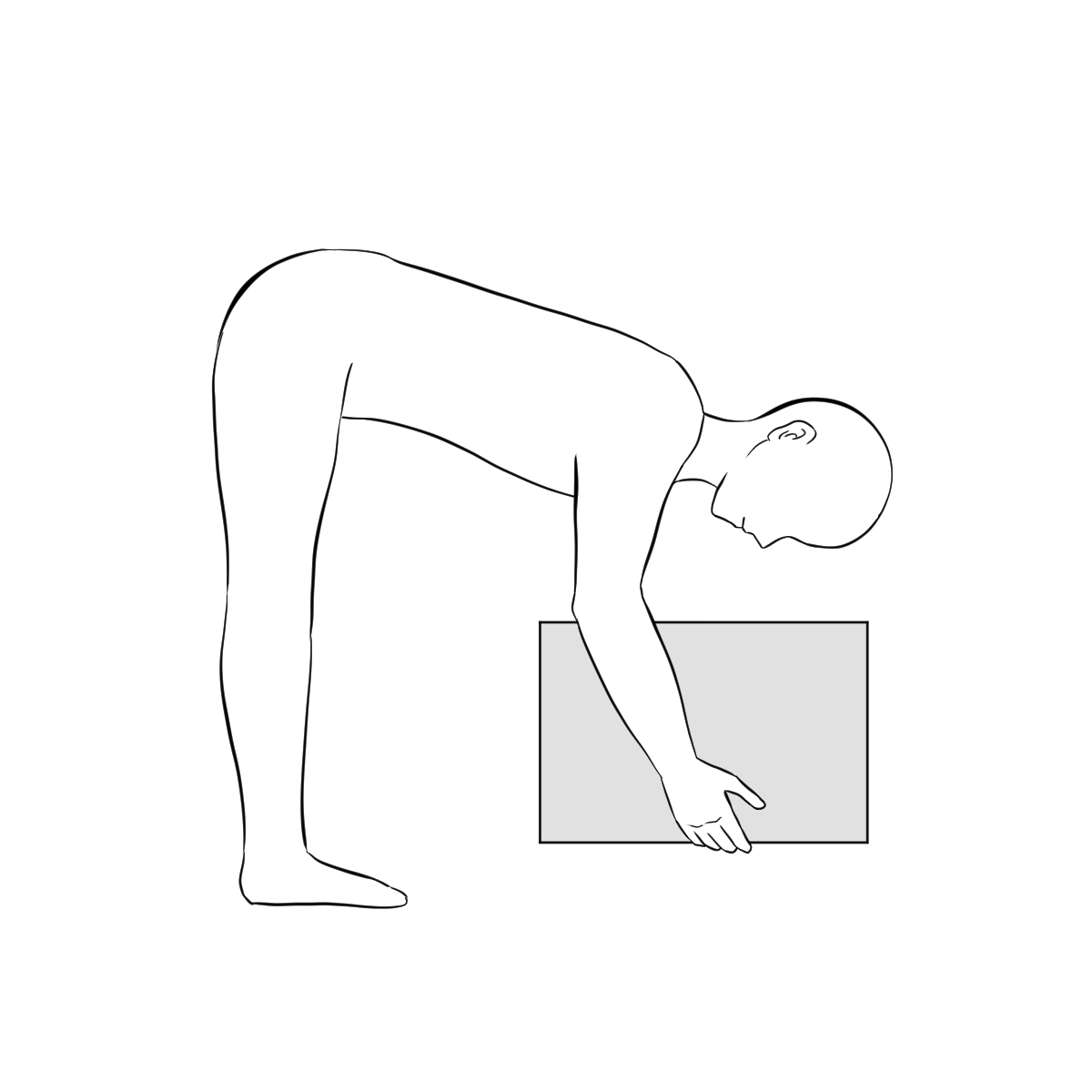

Supplement: Supplementary file 1 [file healthcare-12-02274-s001.zip › healthcare-3279036-supplementary/healthcare20241108_Postures/1-2_TIFF.tif]

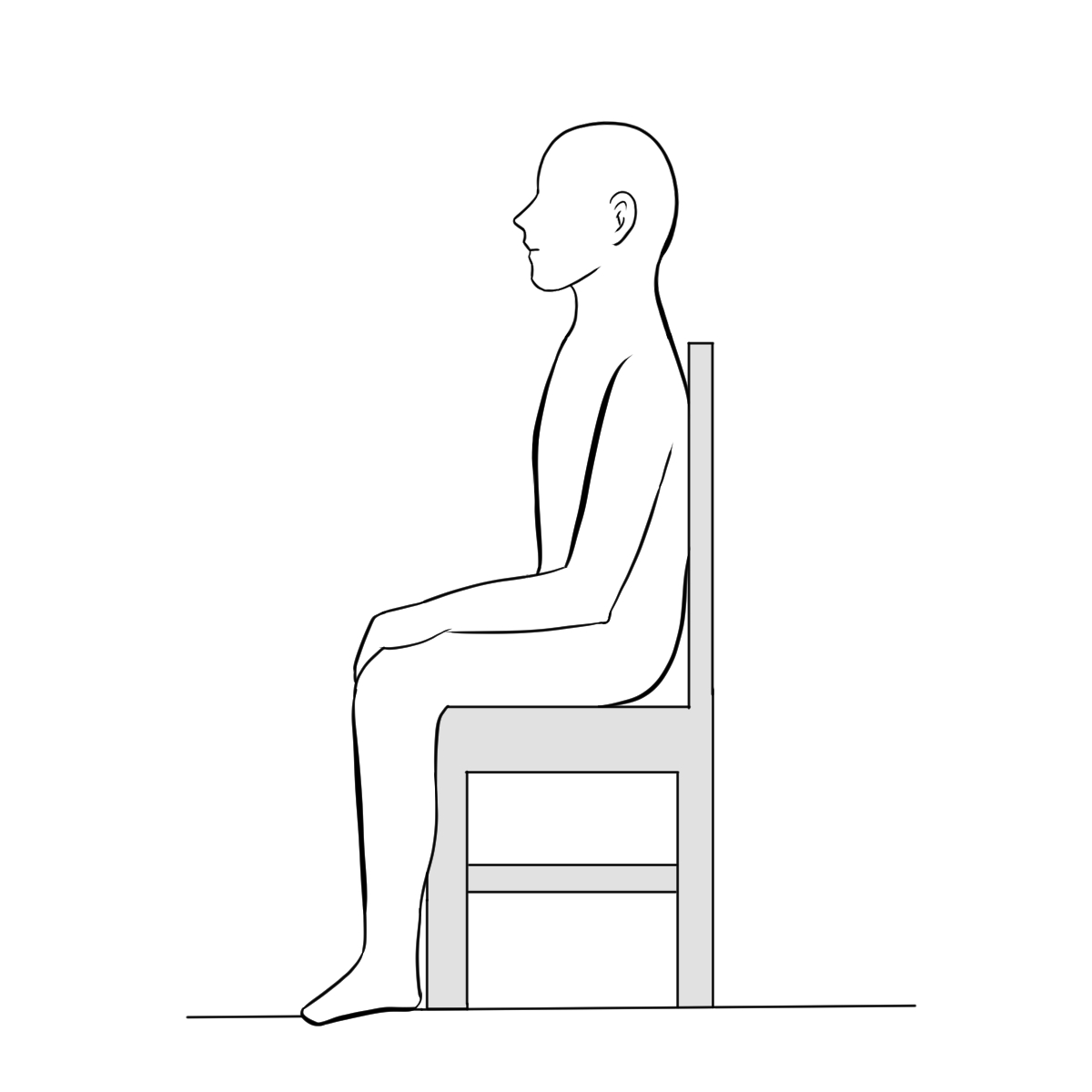

Supplement: Supplementary file 1 [file healthcare-12-02274-s001.zip › healthcare-3279036-supplementary/healthcare20241108_Postures/2-1-1.jpg]

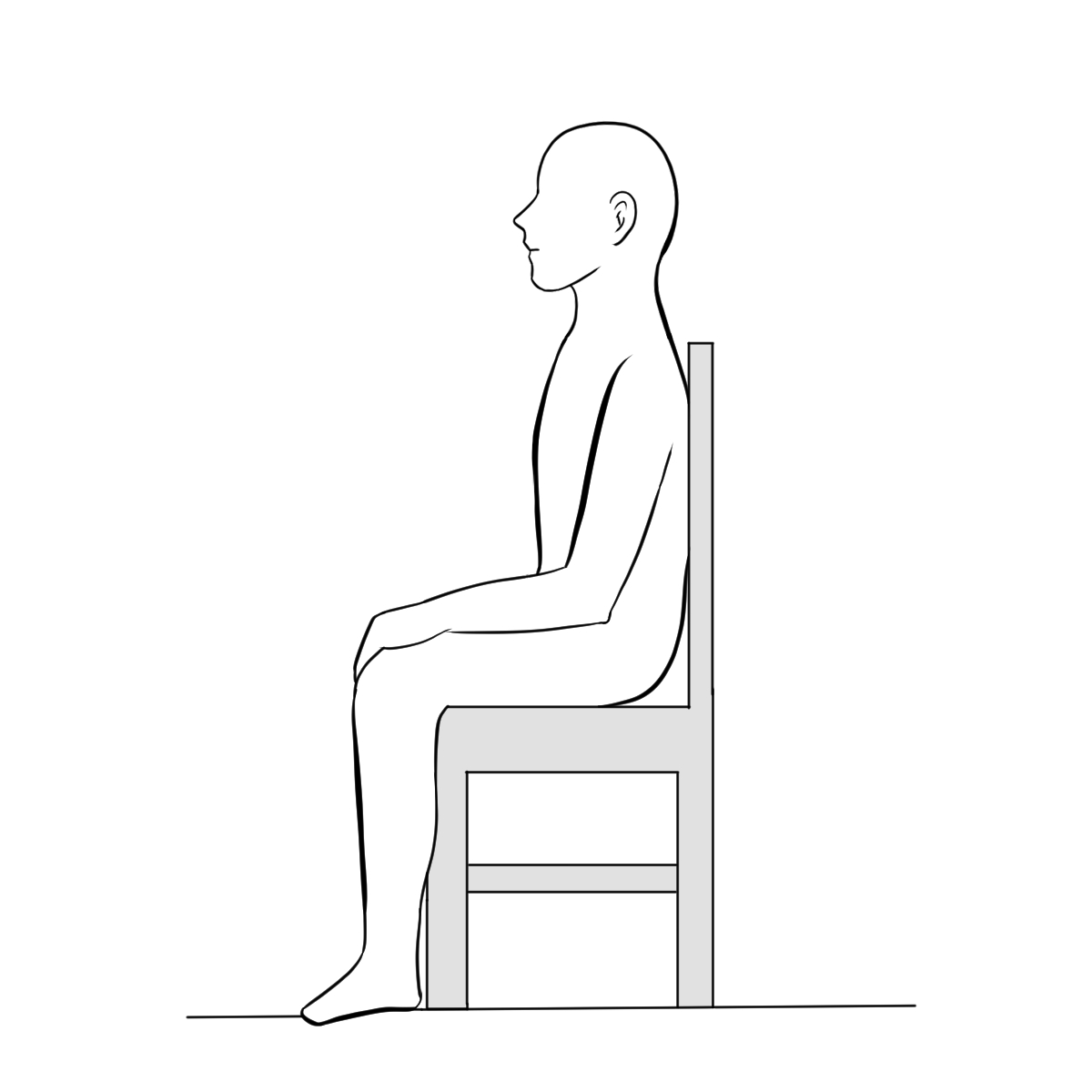

Supplement: Supplementary file 1 [file healthcare-12-02274-s001.zip › healthcare-3279036-supplementary/healthcare20241108_Postures/2-1-1_TIFF.tif]

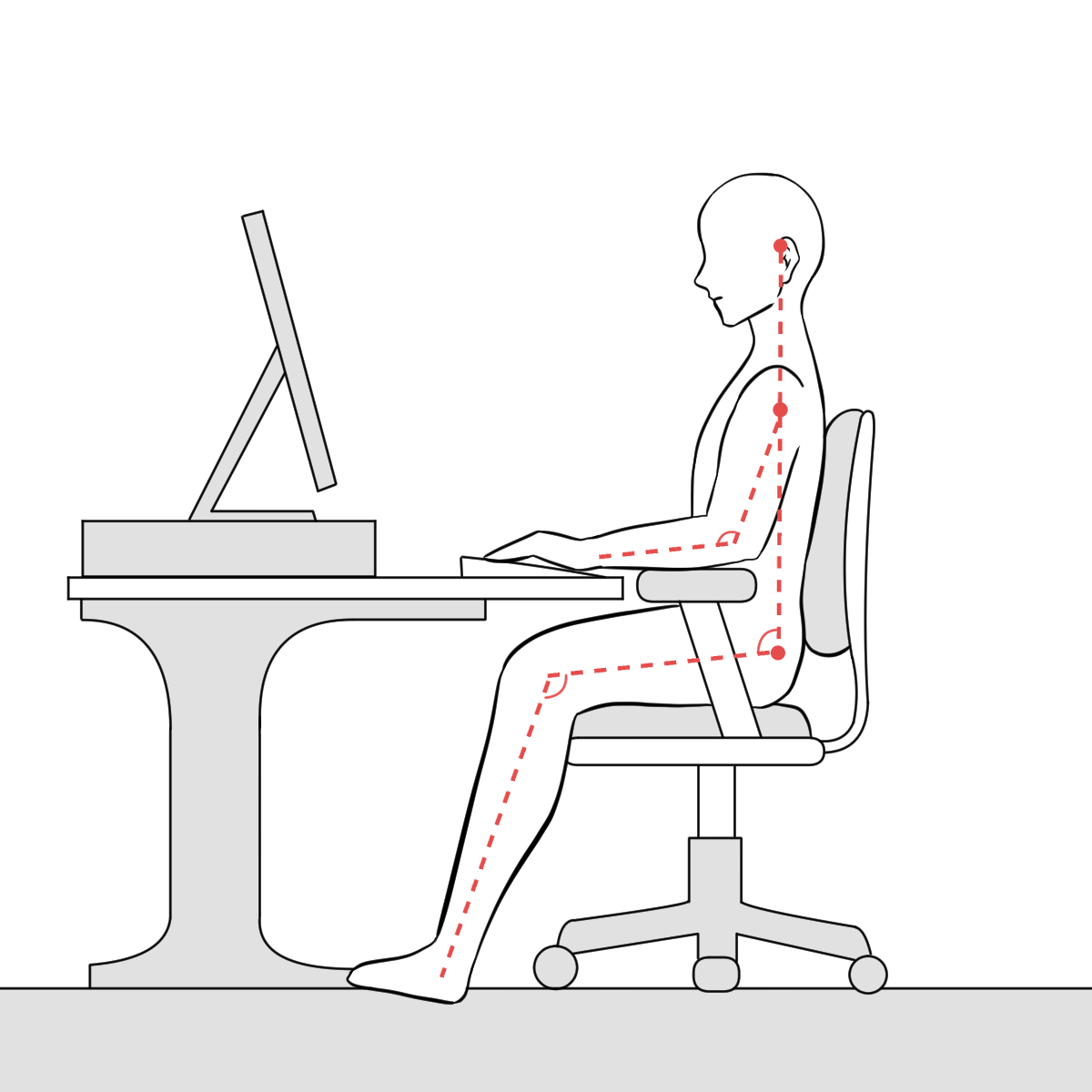

Supplement: Supplementary file 1 [file healthcare-12-02274-s001.zip › healthcare-3279036-supplementary/healthcare20241108_Postures/2-1-2.jpg]

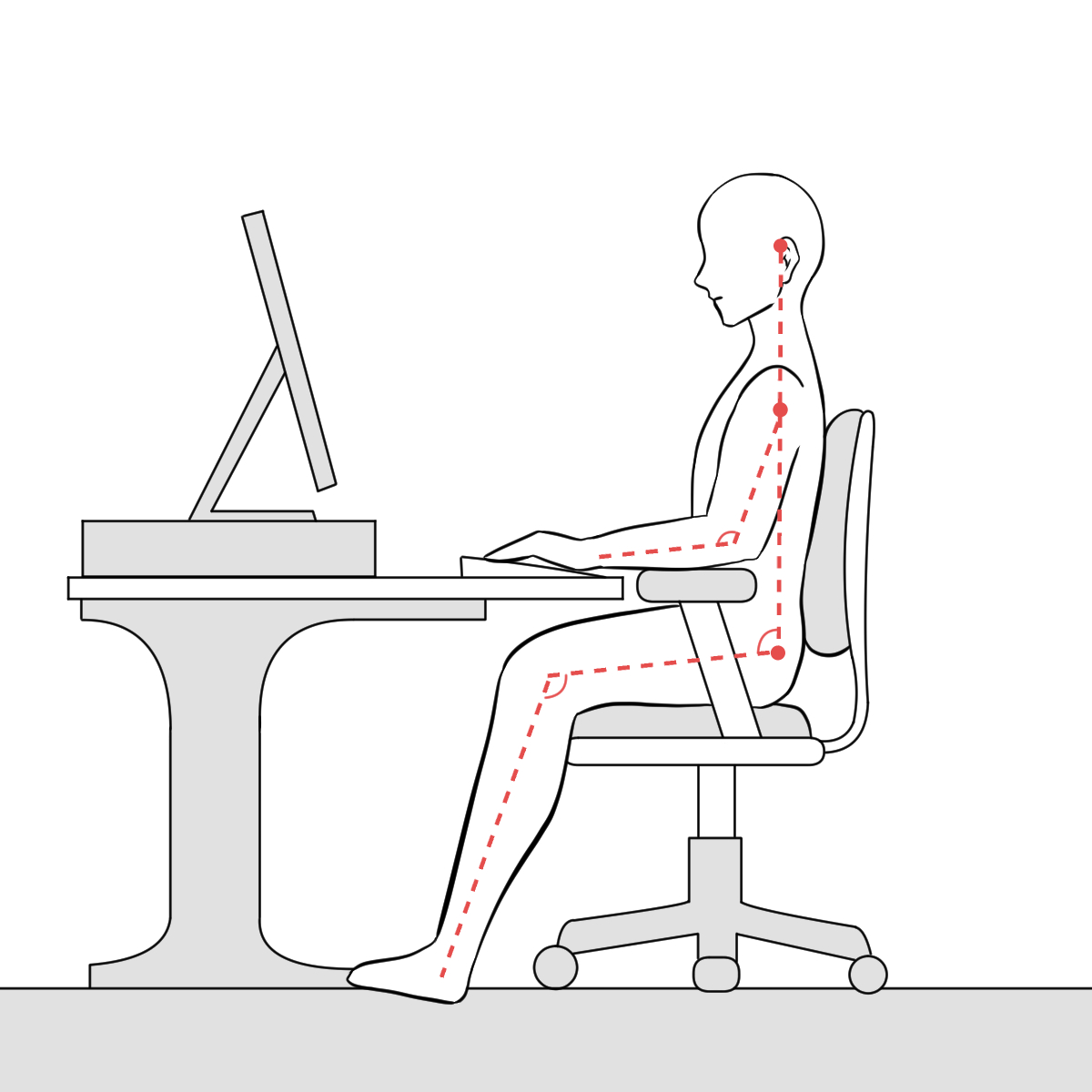

Supplement: Supplementary file 1 [file healthcare-12-02274-s001.zip › healthcare-3279036-supplementary/healthcare20241108_Postures/2-1-2_TIFF.tif]

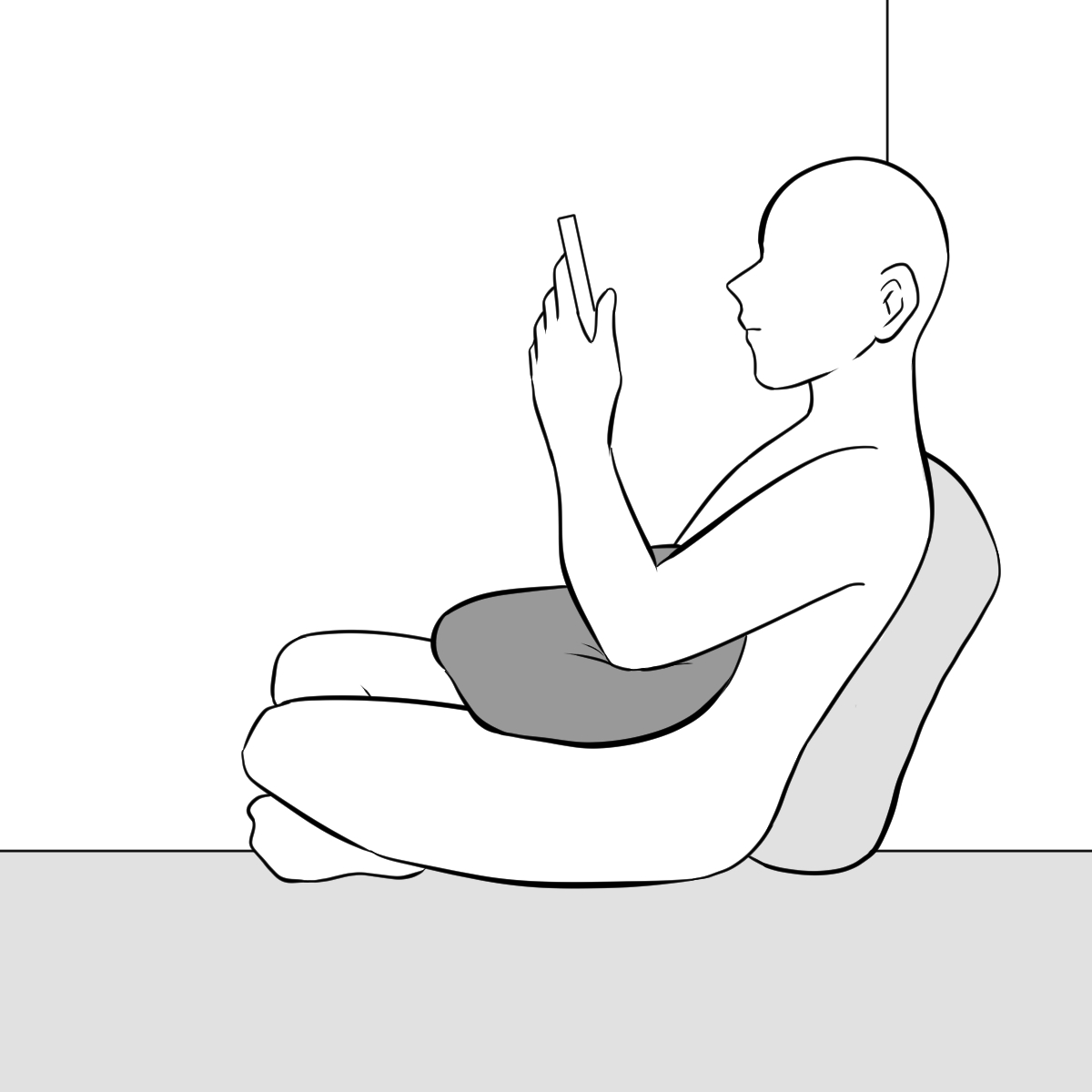

Supplement: Supplementary file 1 [file healthcare-12-02274-s001.zip › healthcare-3279036-supplementary/healthcare20241108_Postures/2-1-3.jpg]

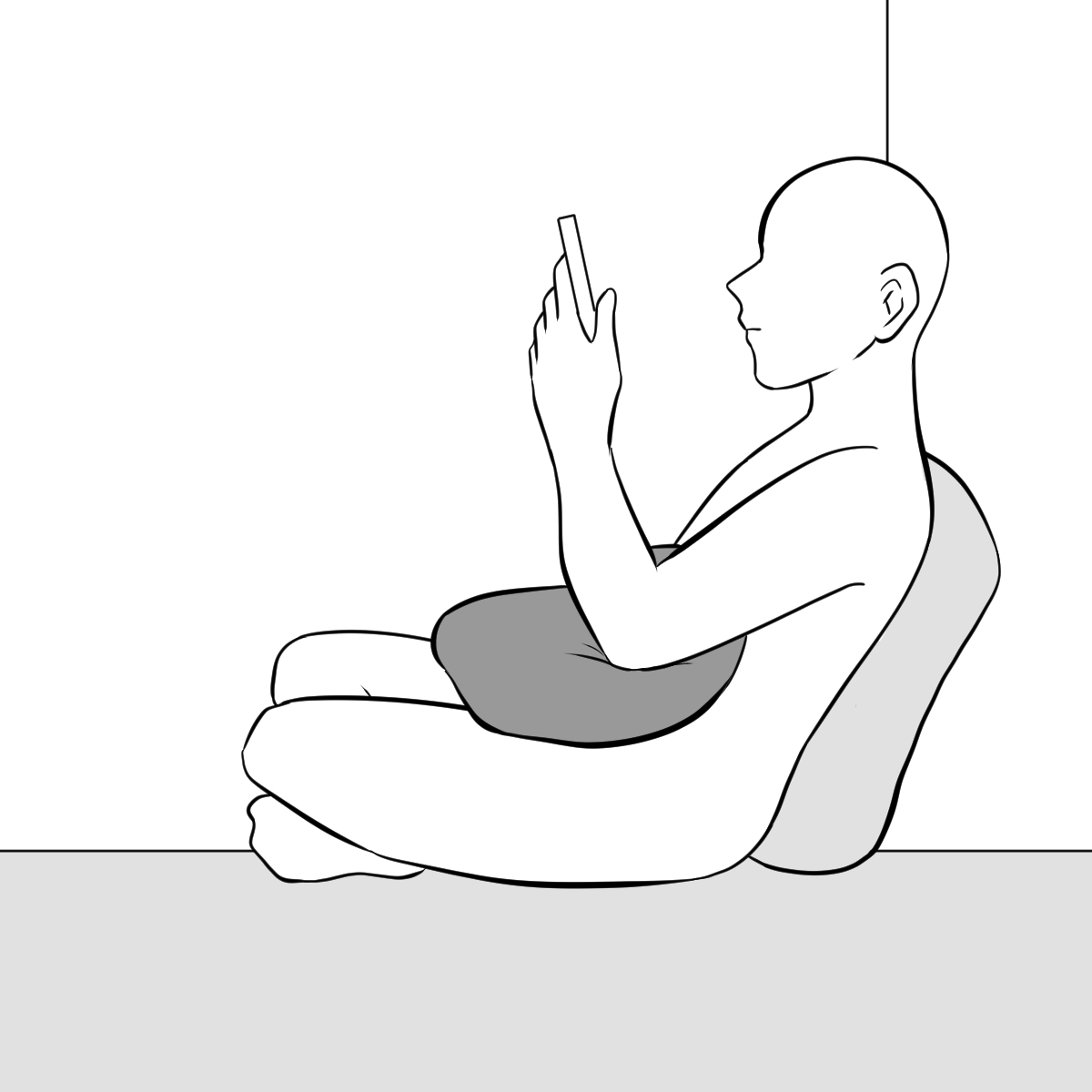

Supplement: Supplementary file 1 [file healthcare-12-02274-s001.zip › healthcare-3279036-supplementary/healthcare20241108_Postures/2-1-3_TIFF.tif]

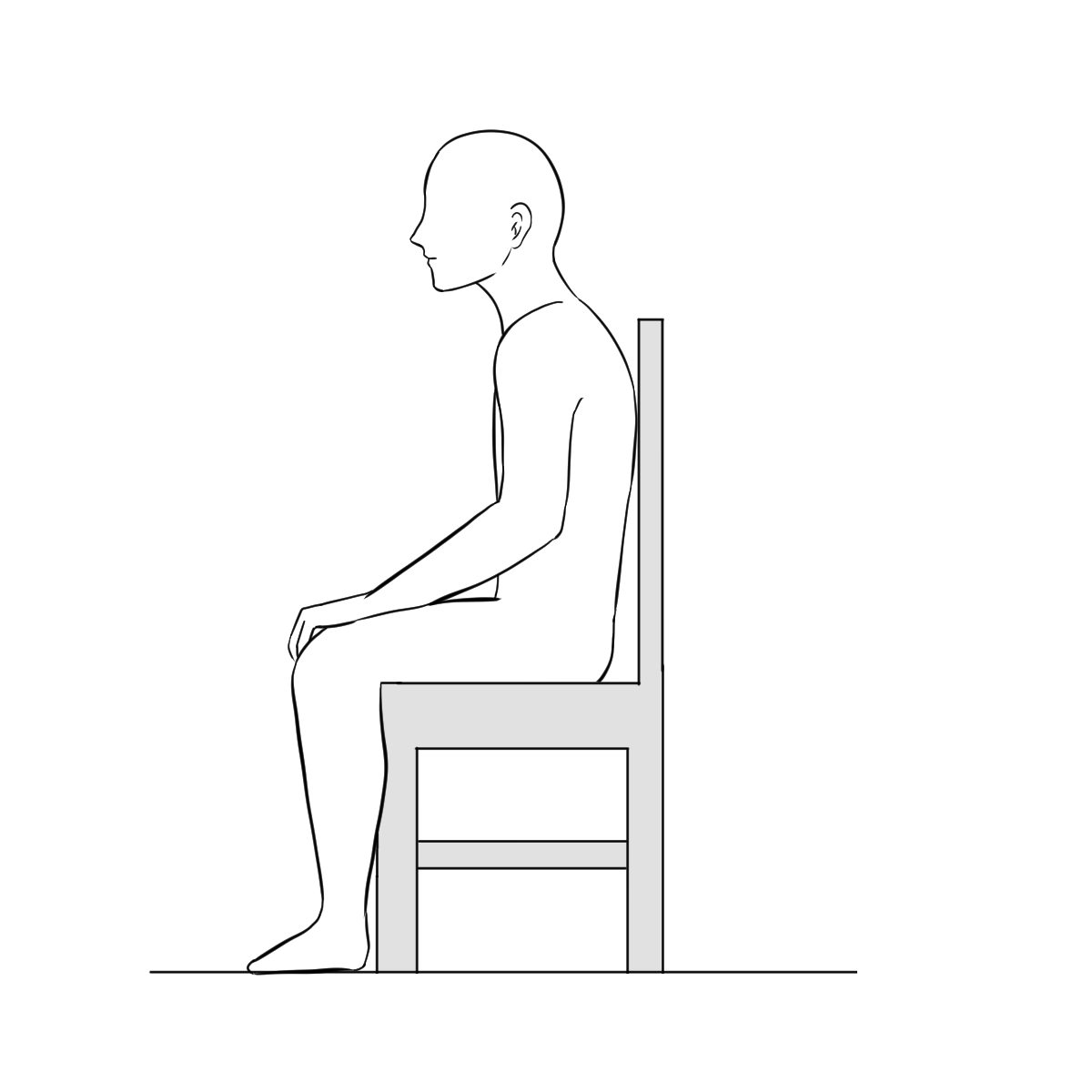

Supplement: Supplementary file 1 [file healthcare-12-02274-s001.zip › healthcare-3279036-supplementary/healthcare20241108_Postures/2-2-1.jpg]

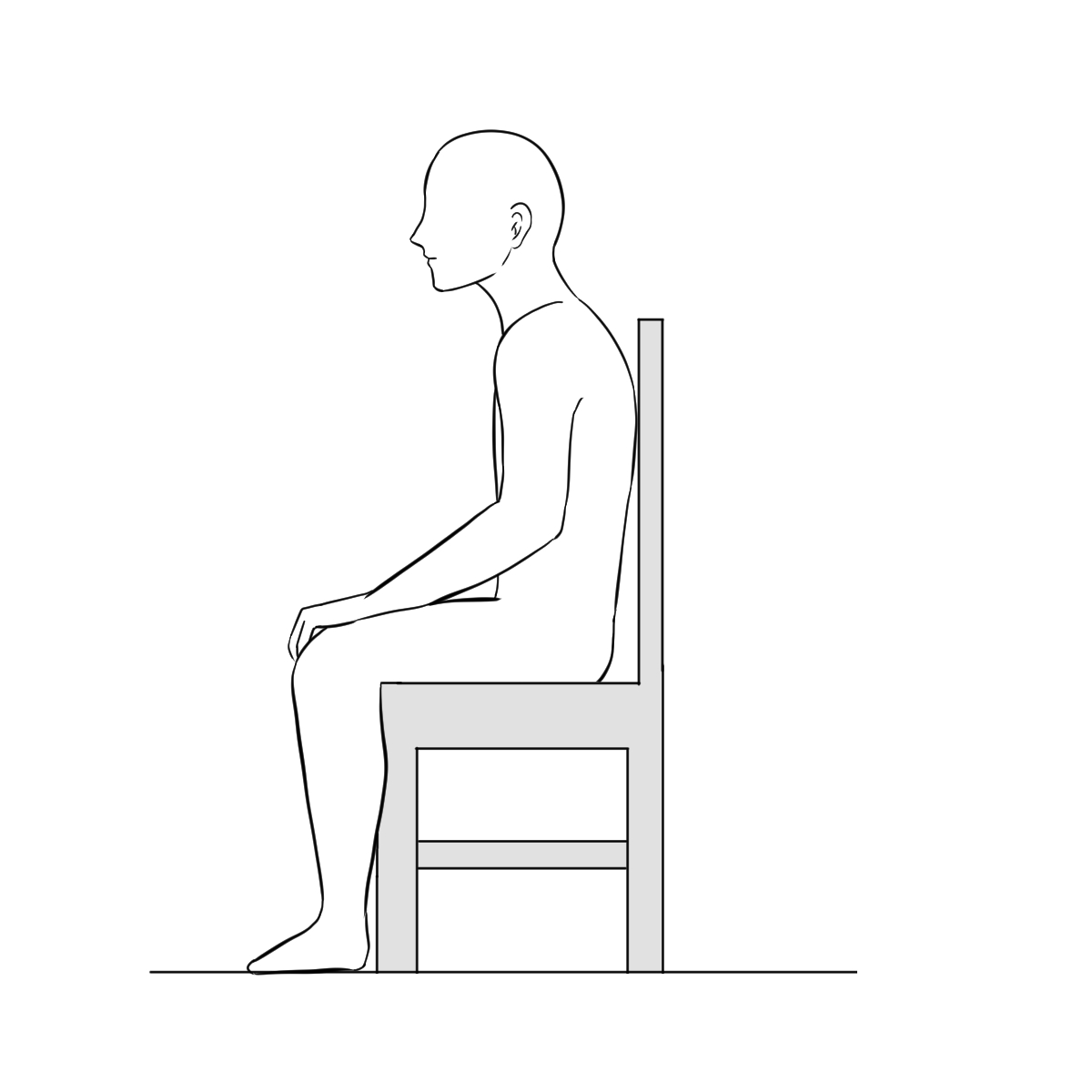

Supplement: Supplementary file 1 [file healthcare-12-02274-s001.zip › healthcare-3279036-supplementary/healthcare20241108_Postures/2-2-1_TIFF.tif]

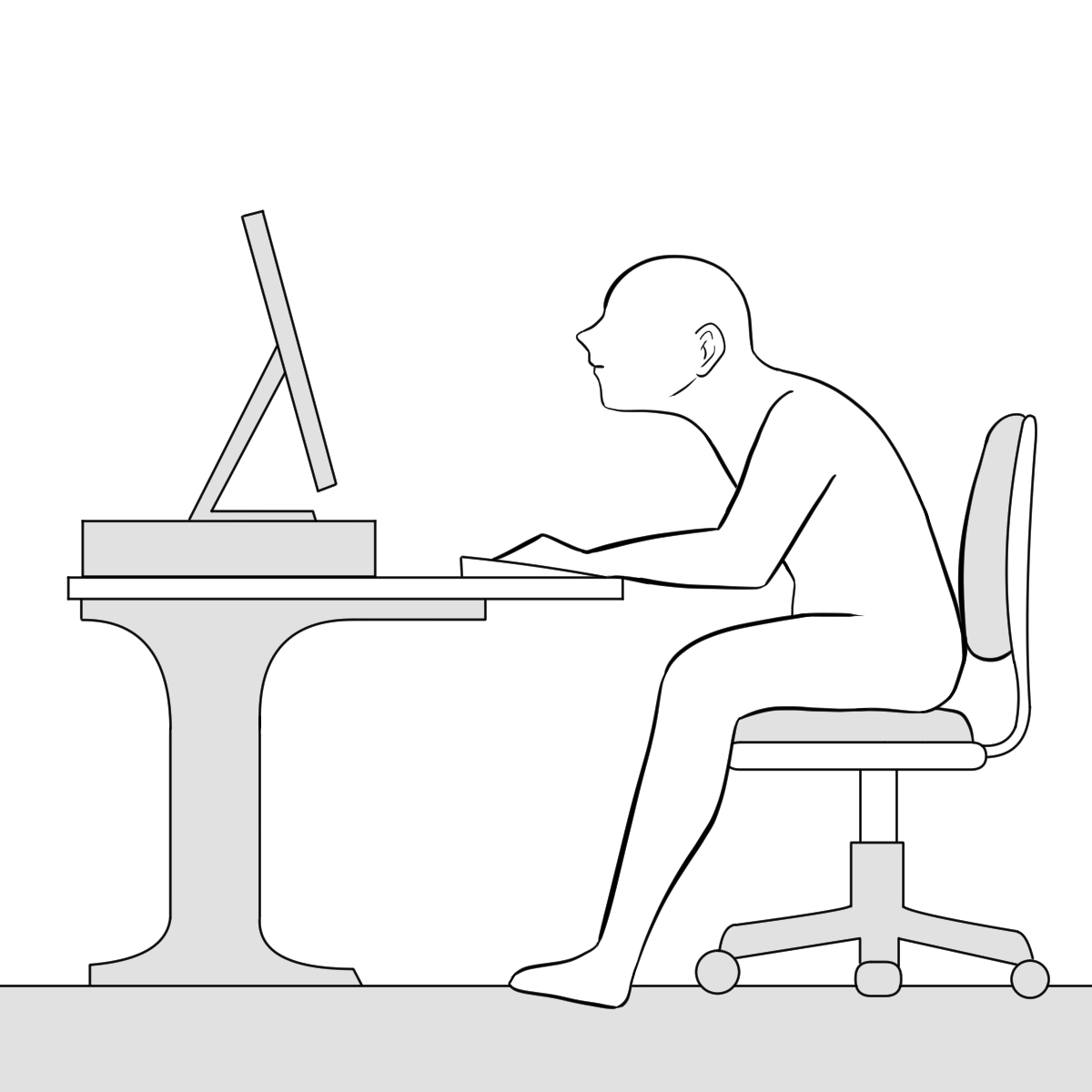

Supplement: Supplementary file 1 [file healthcare-12-02274-s001.zip › healthcare-3279036-supplementary/healthcare20241108_Postures/2-2-2.jpg]

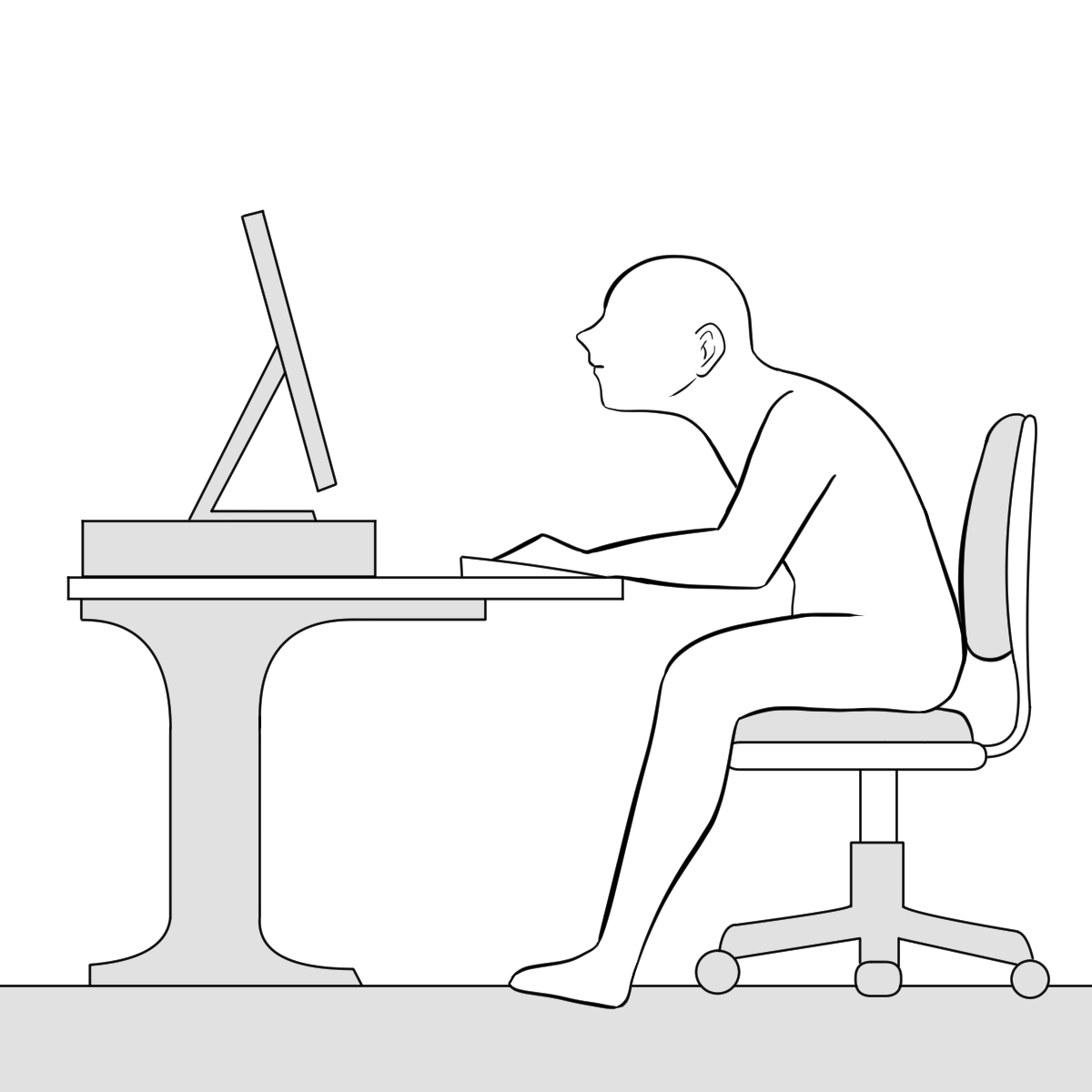

Supplement: Supplementary file 1 [file healthcare-12-02274-s001.zip › healthcare-3279036-supplementary/healthcare20241108_Postures/2-2-2_TIFF.tif]

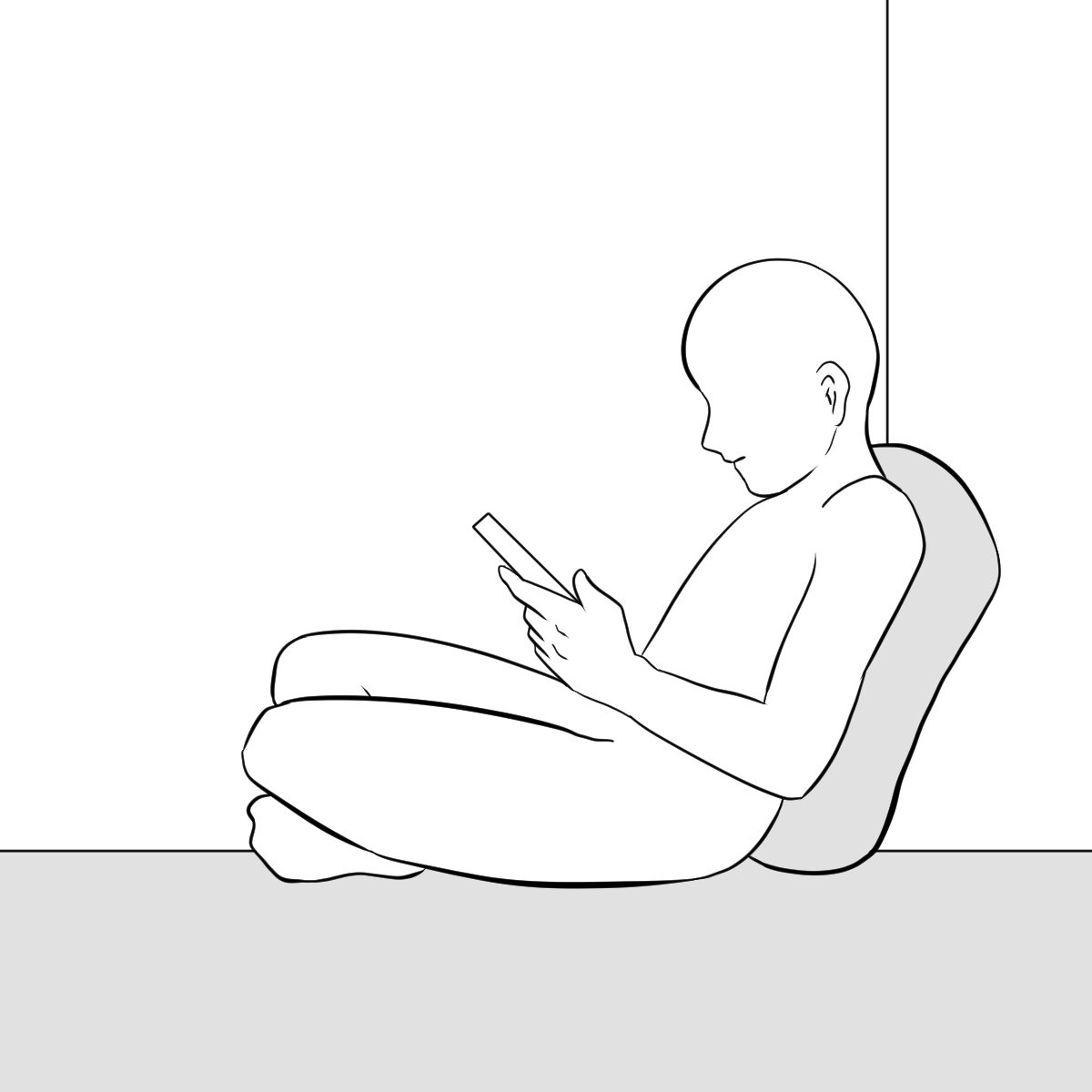

Supplement: Supplementary file 1 [file healthcare-12-02274-s001.zip › healthcare-3279036-supplementary/healthcare20241108_Postures/2-2-3.jpg]

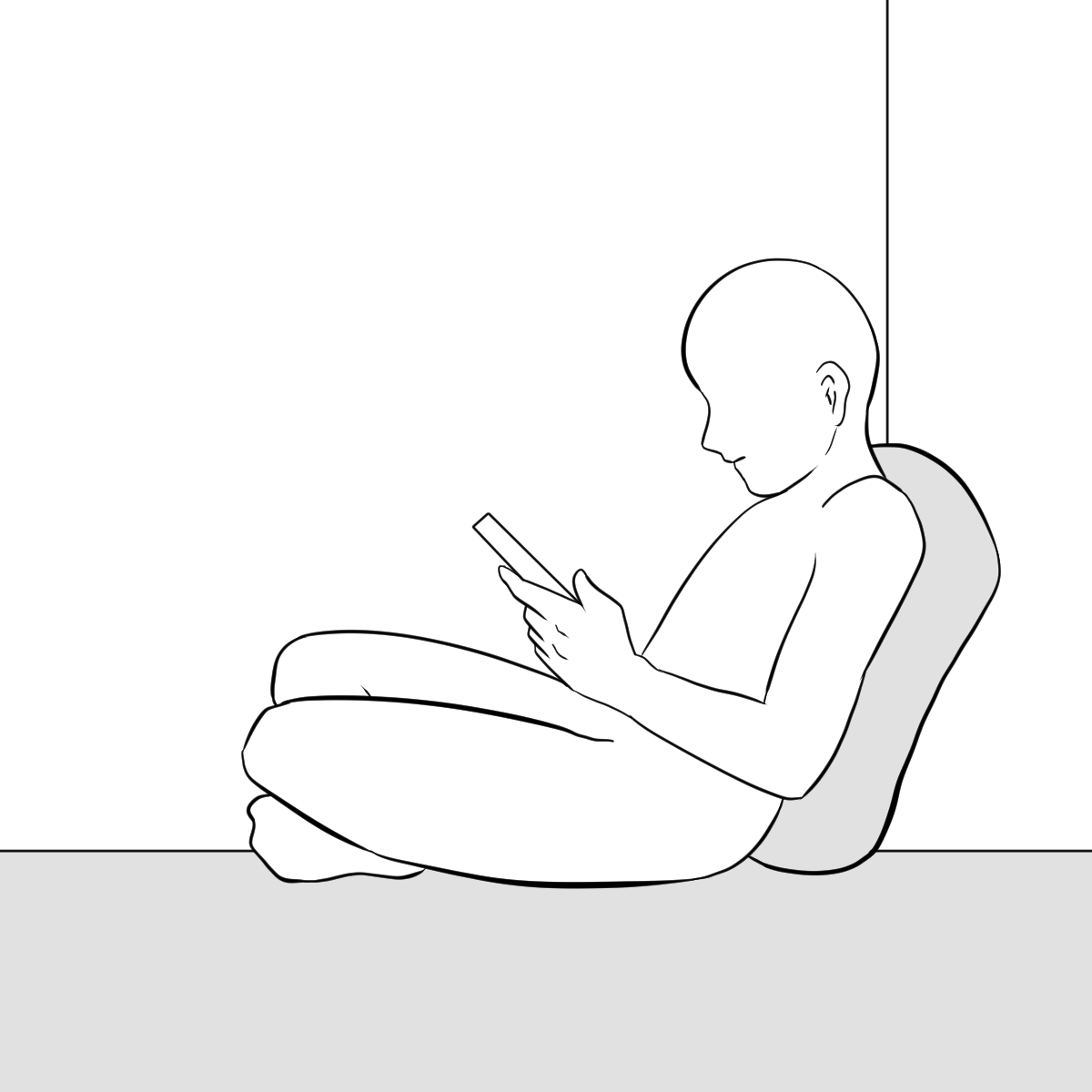

Supplement: Supplementary file 1 [file healthcare-12-02274-s001.zip › healthcare-3279036-supplementary/healthcare20241108_Postures/2-2-3_TIFF.tif]

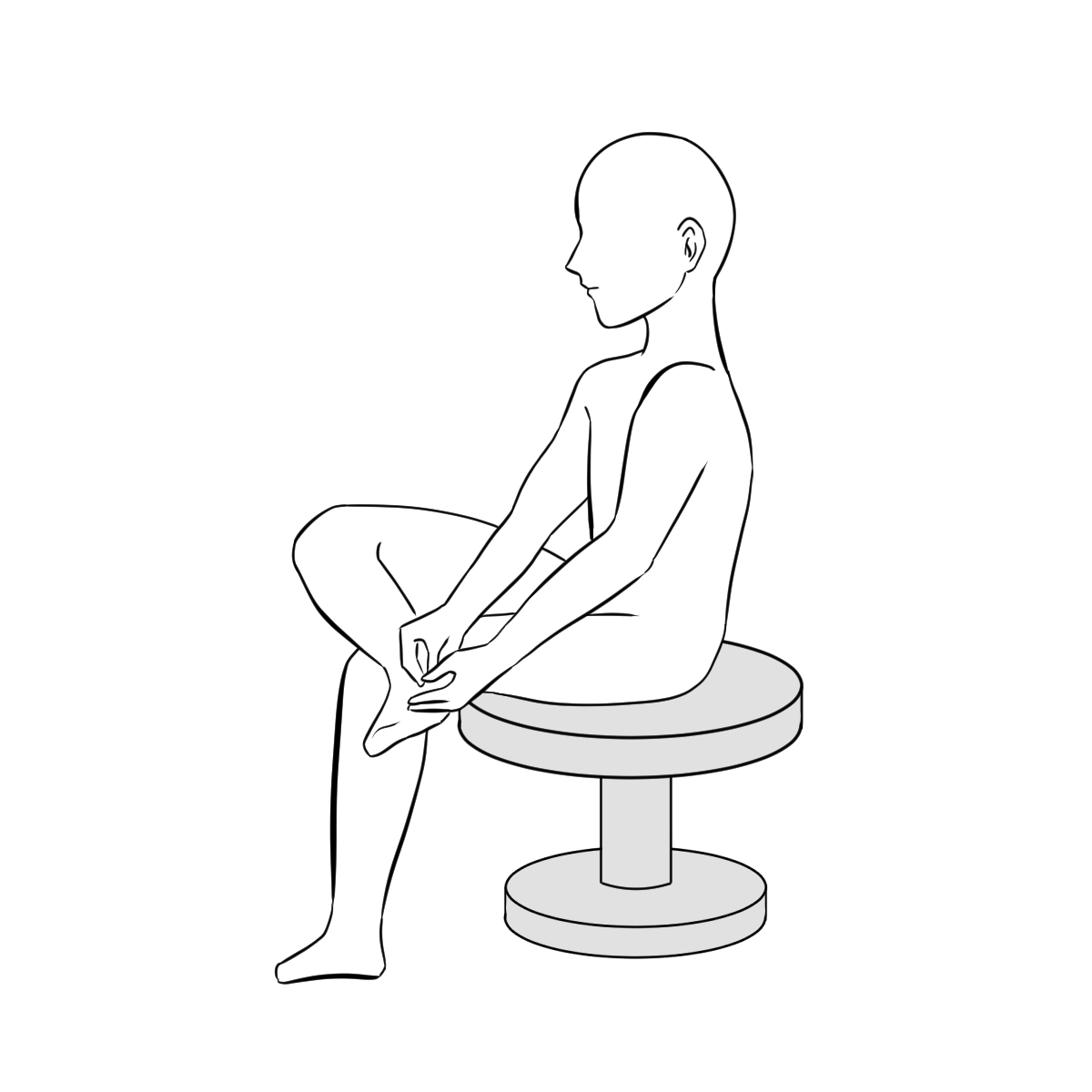

Supplement: Supplementary file 1 [file healthcare-12-02274-s001.zip › healthcare-3279036-supplementary/healthcare20241108_Postures/3-1-1.jpg]

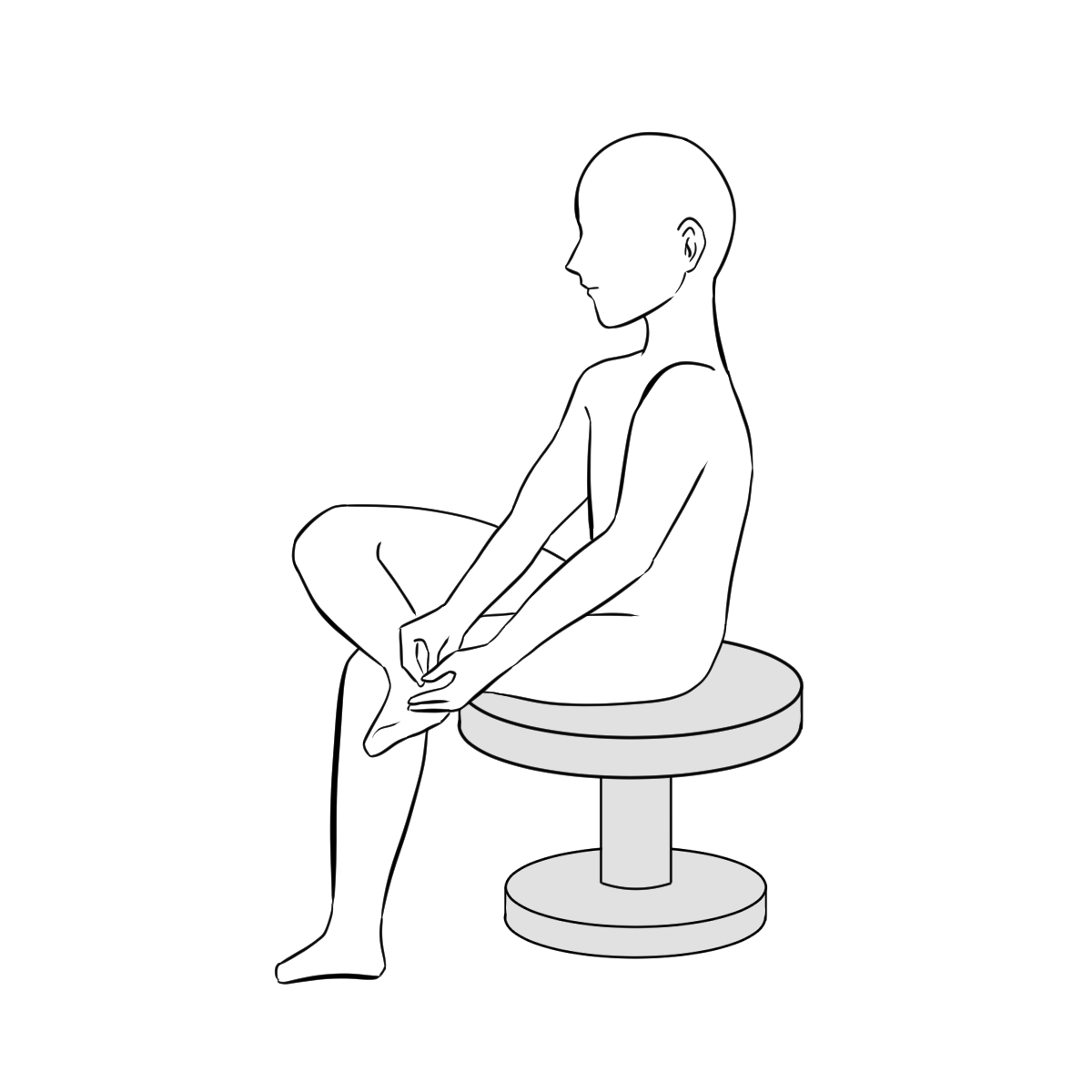

Supplement: Supplementary file 1 [file healthcare-12-02274-s001.zip › healthcare-3279036-supplementary/healthcare20241108_Postures/3-1-1_TIFF.tif]

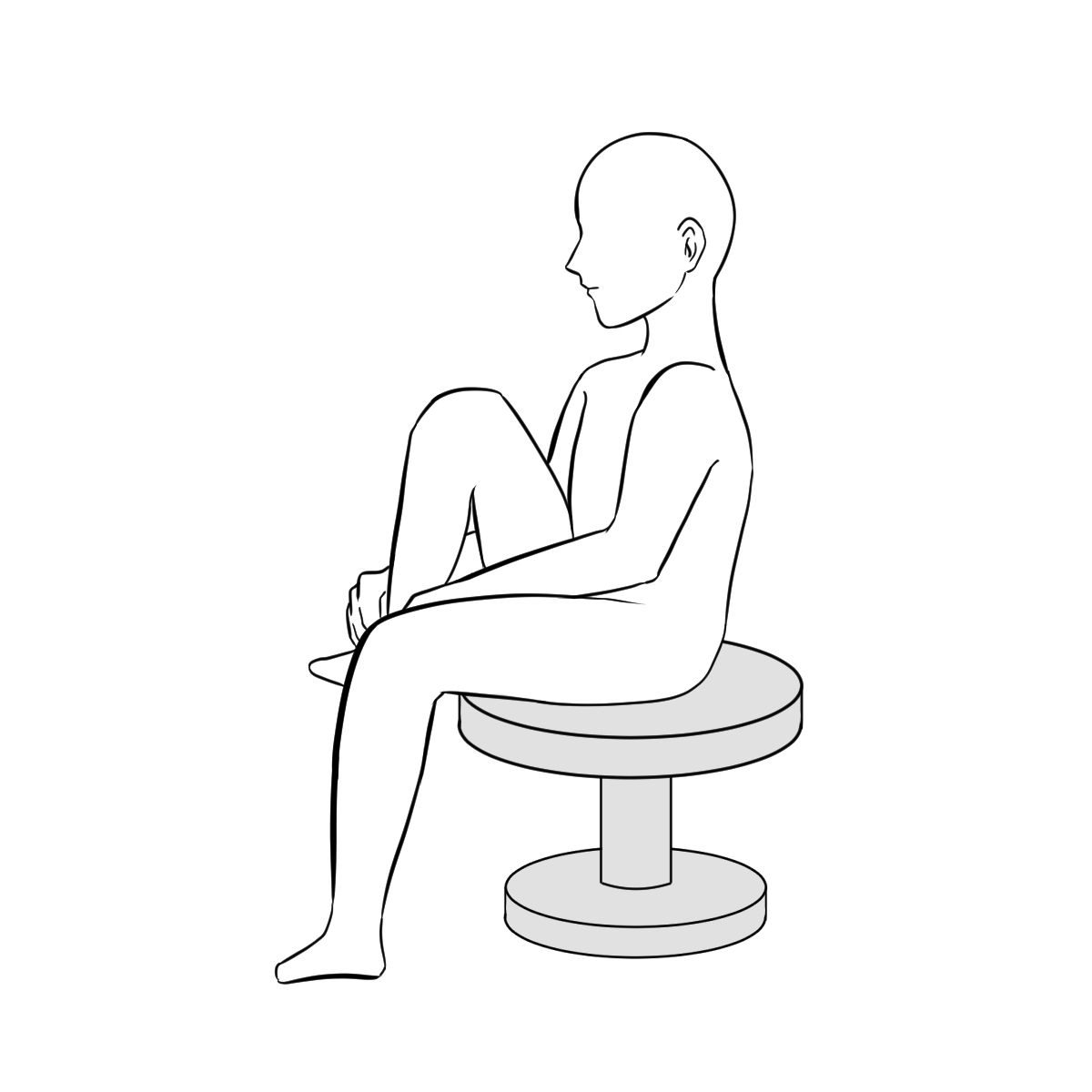

Supplement: Supplementary file 1 [file healthcare-12-02274-s001.zip › healthcare-3279036-supplementary/healthcare20241108_Postures/3-1-2.jpg]

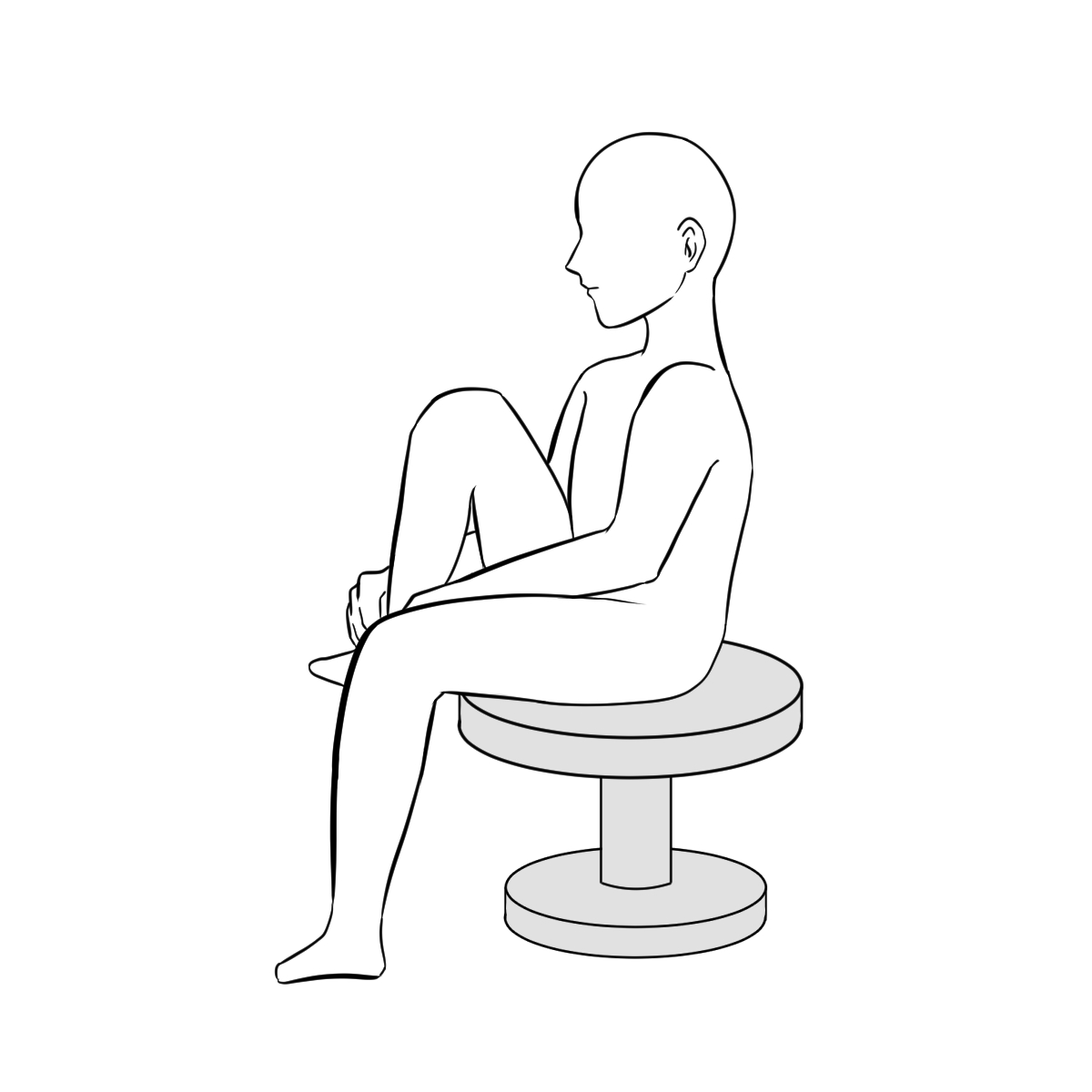

Supplement: Supplementary file 1 [file healthcare-12-02274-s001.zip › healthcare-3279036-supplementary/healthcare20241108_Postures/3-1-2_TIFF.tif]

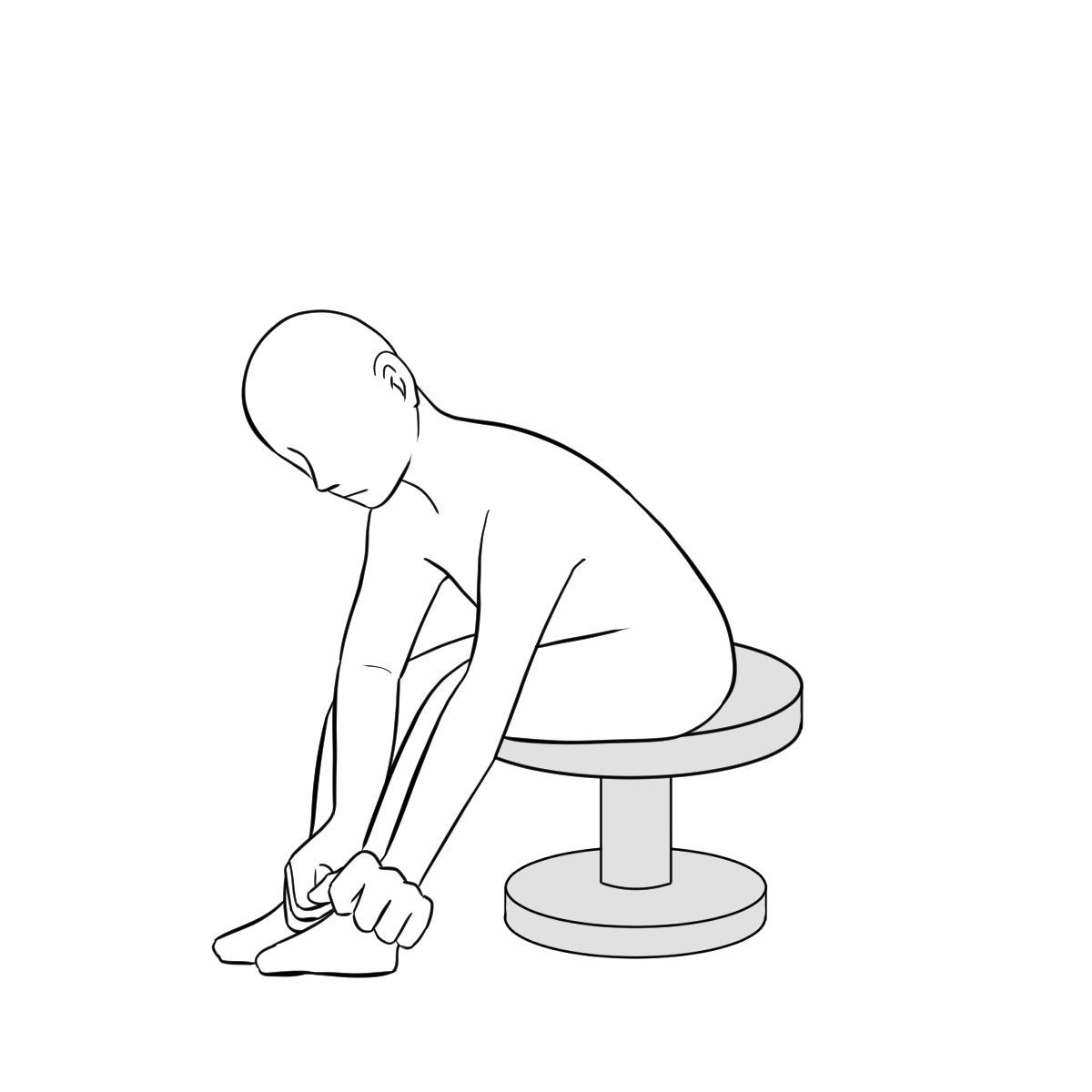

Supplement: Supplementary file 1 [file healthcare-12-02274-s001.zip › healthcare-3279036-supplementary/healthcare20241108_Postures/3-2-1.jpg]

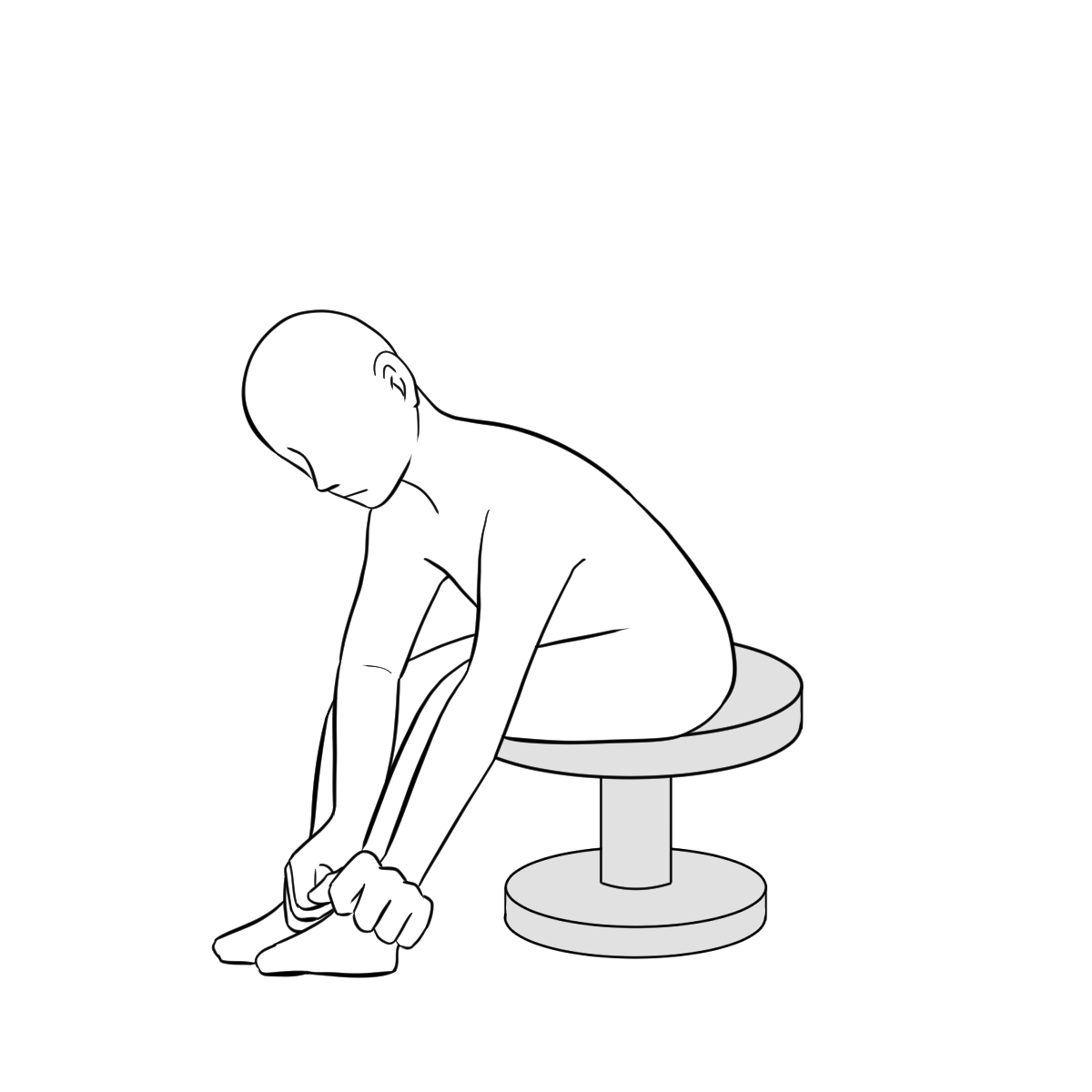

Supplement: Supplementary file 1 [file healthcare-12-02274-s001.zip › healthcare-3279036-supplementary/healthcare20241108_Postures/3-2-1_TIFF.tif]

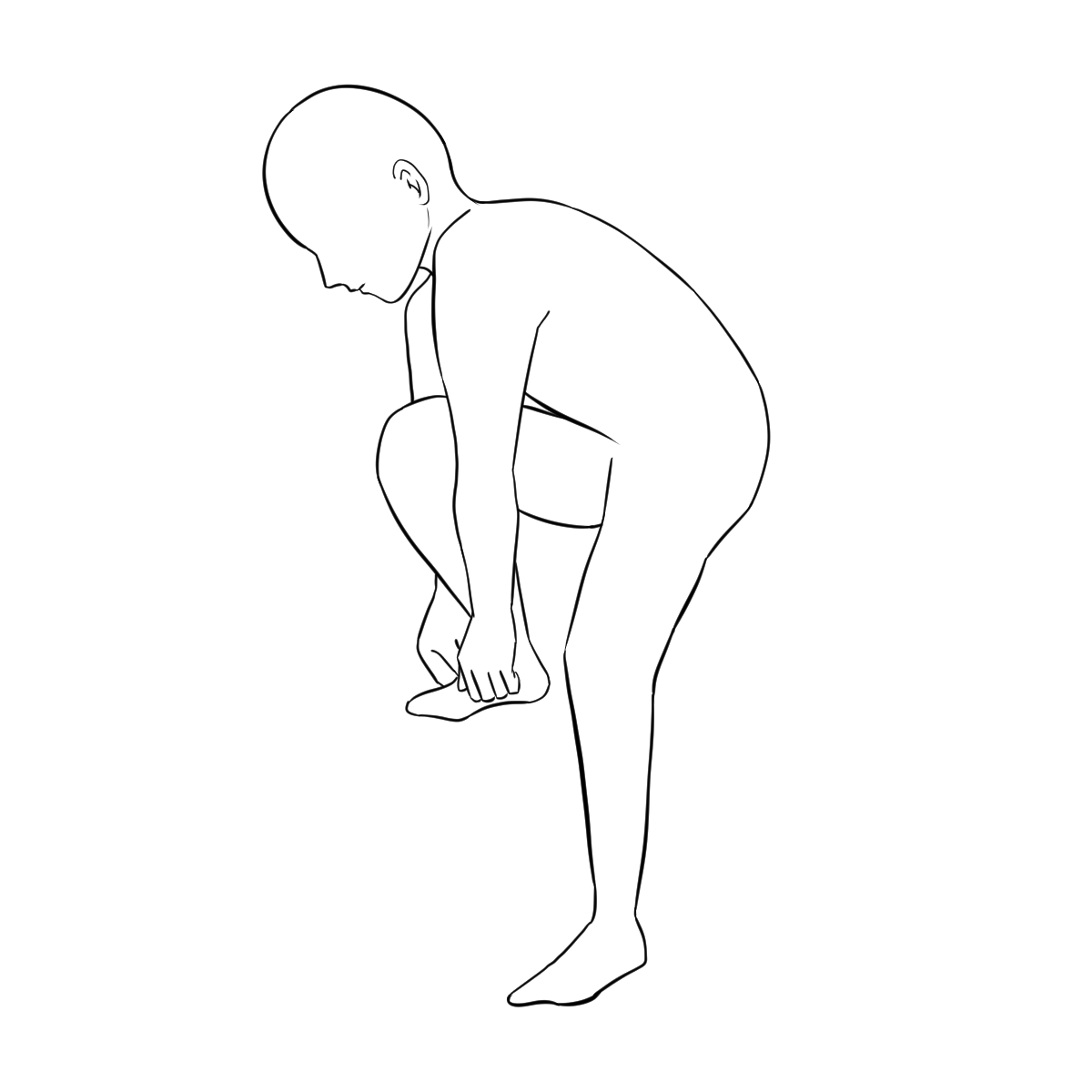

Supplement: Supplementary file 1 [file healthcare-12-02274-s001.zip › healthcare-3279036-supplementary/healthcare20241108_Postures/3-2-2.jpg]

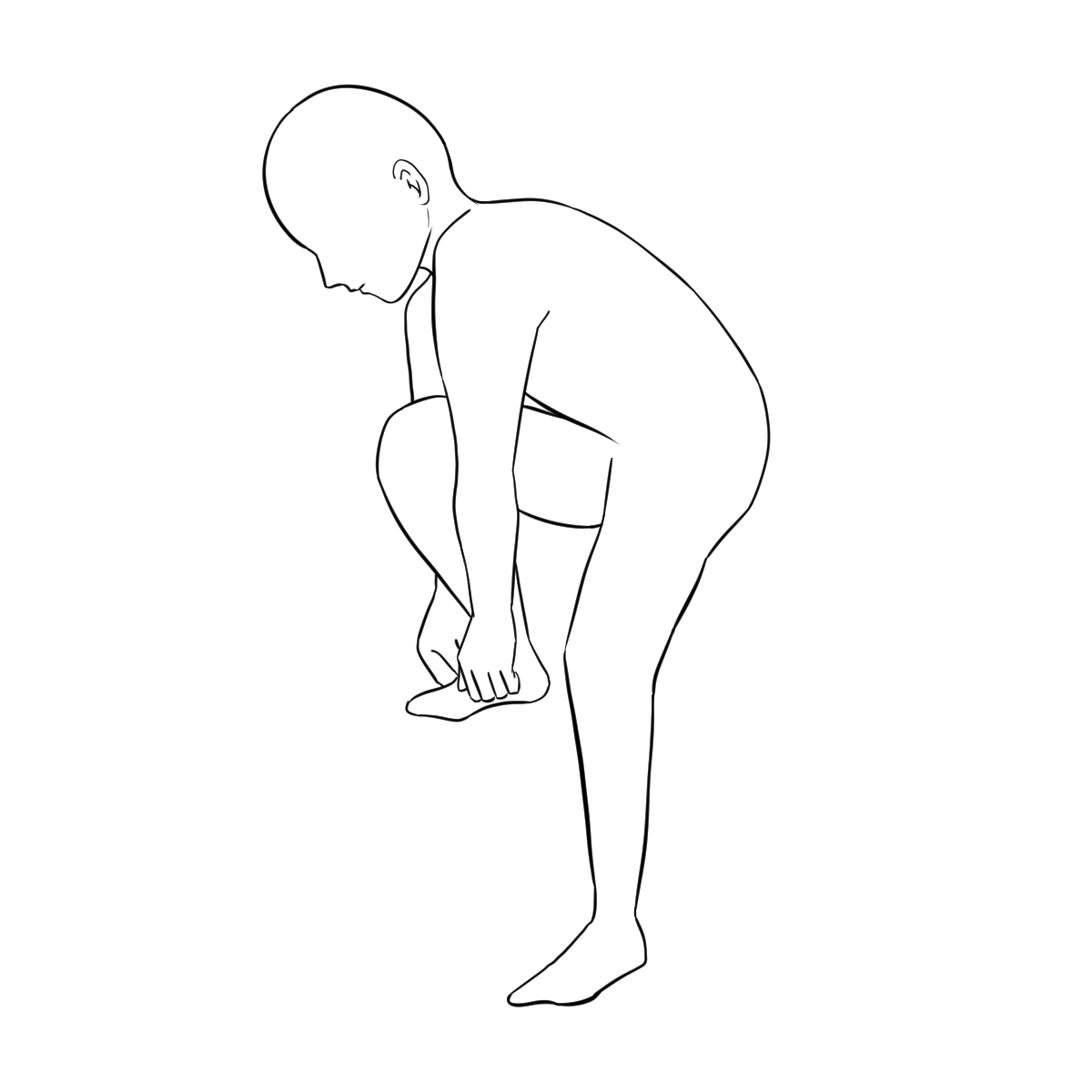

Supplement: Supplementary file 1 [file healthcare-12-02274-s001.zip › healthcare-3279036-supplementary/healthcare20241108_Postures/3-2-2_TIFF.tif]

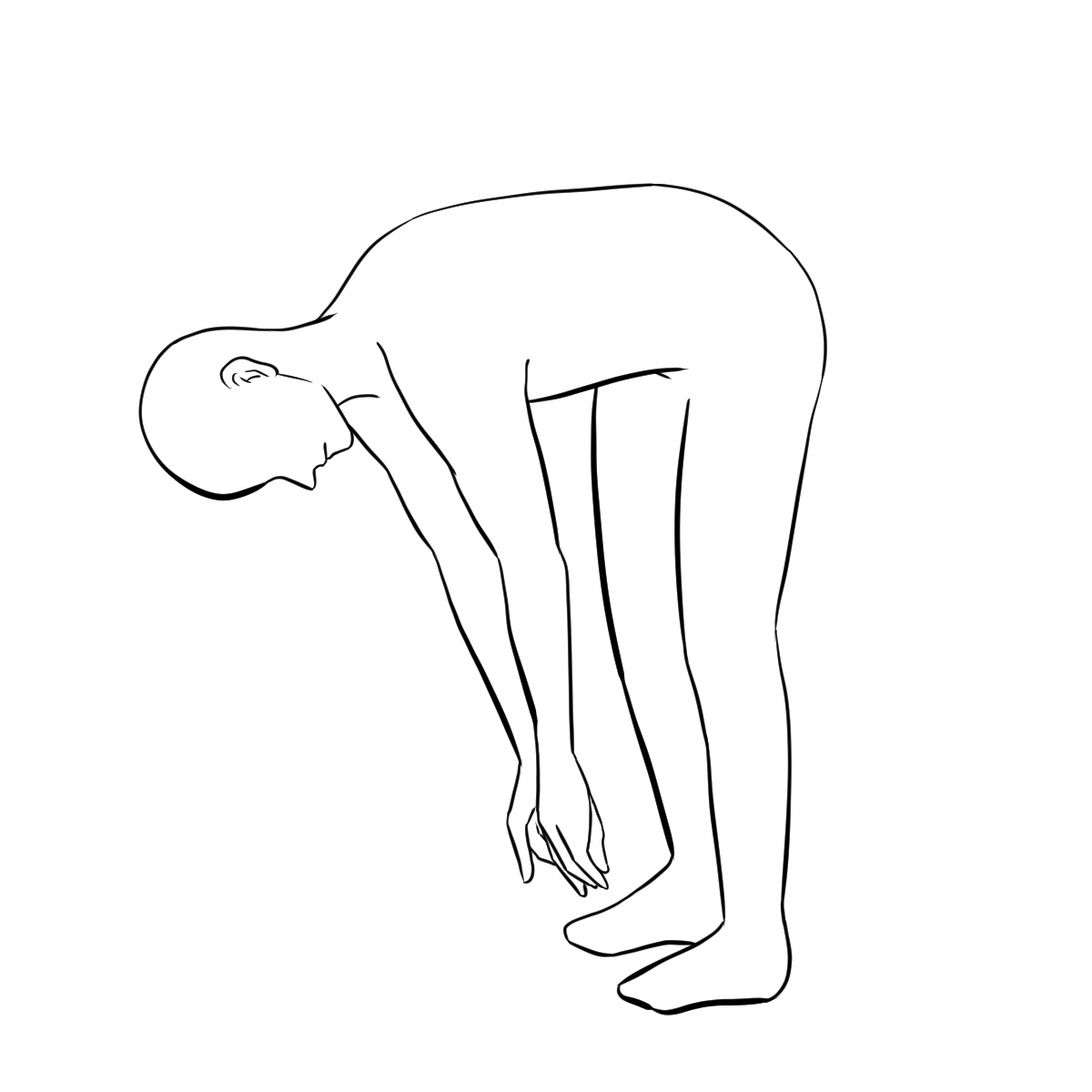

Supplement: Supplementary file 1 [file healthcare-12-02274-s001.zip › healthcare-3279036-supplementary/healthcare20241108_Postures/3-2-3.jpg]

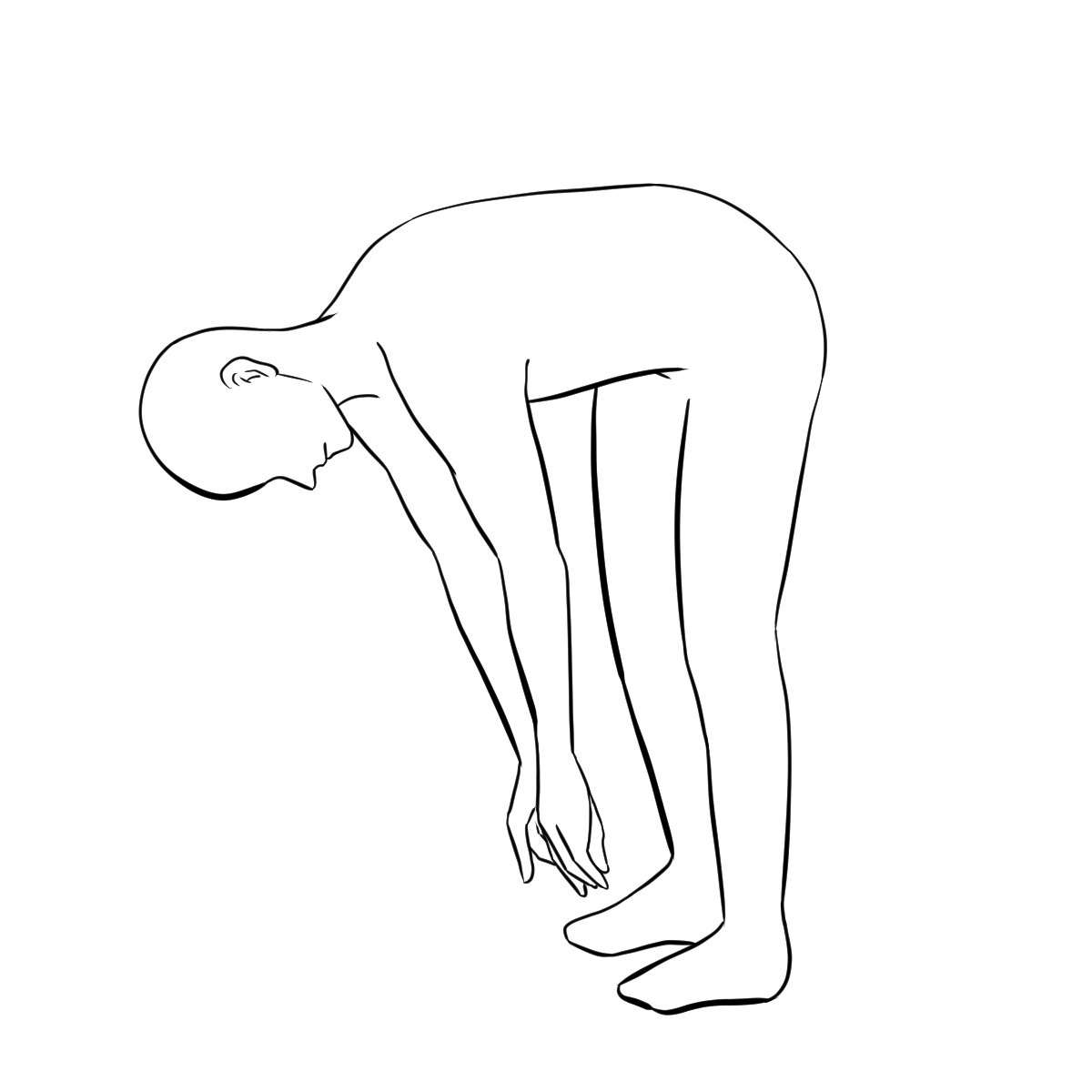

Supplement: Supplementary file 1 [file healthcare-12-02274-s001.zip › healthcare-3279036-supplementary/healthcare20241108_Postures/3-2-3_TIFF.tif]

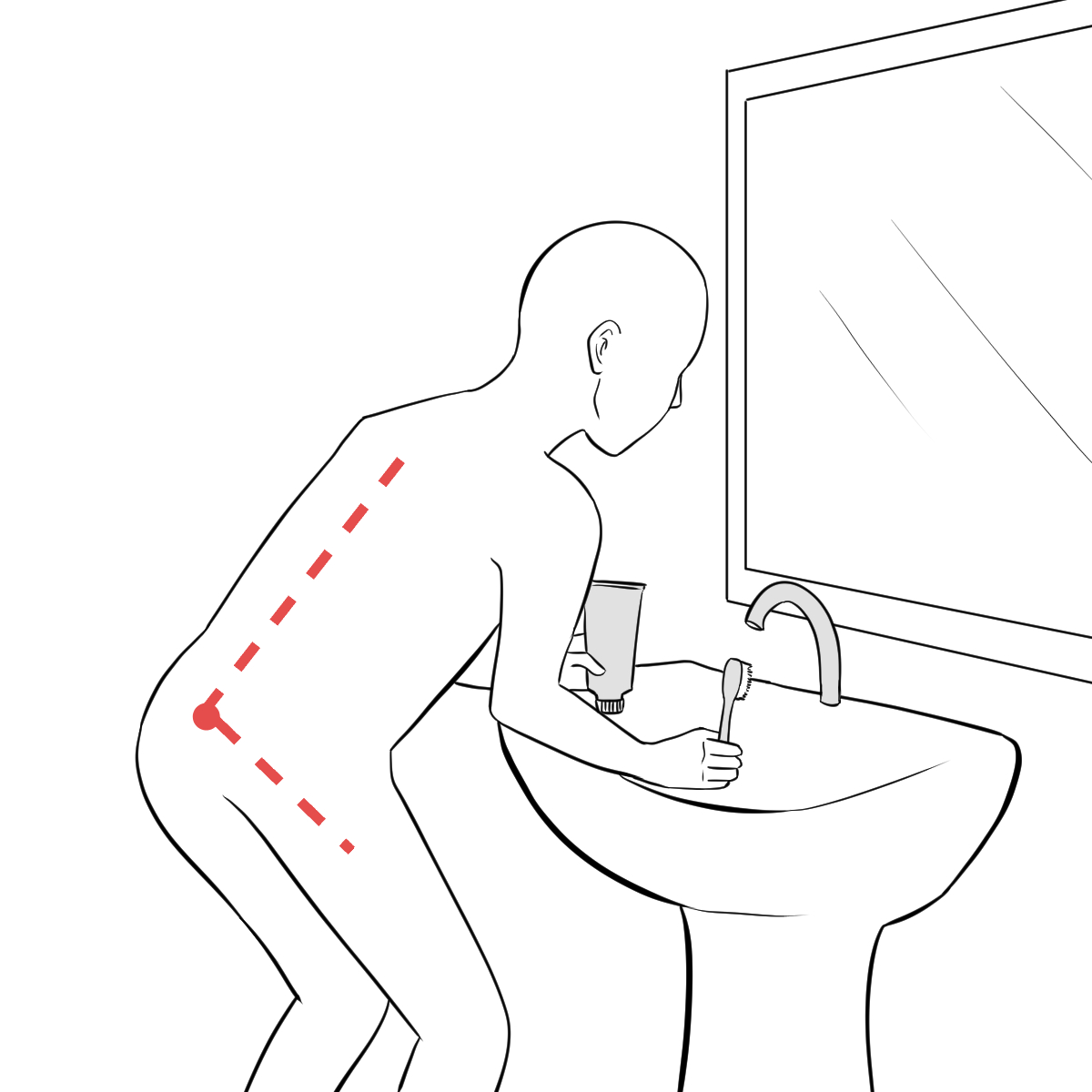

Supplement: Supplementary file 1 [file healthcare-12-02274-s001.zip › healthcare-3279036-supplementary/healthcare20241108_Postures/4-1.jpg]

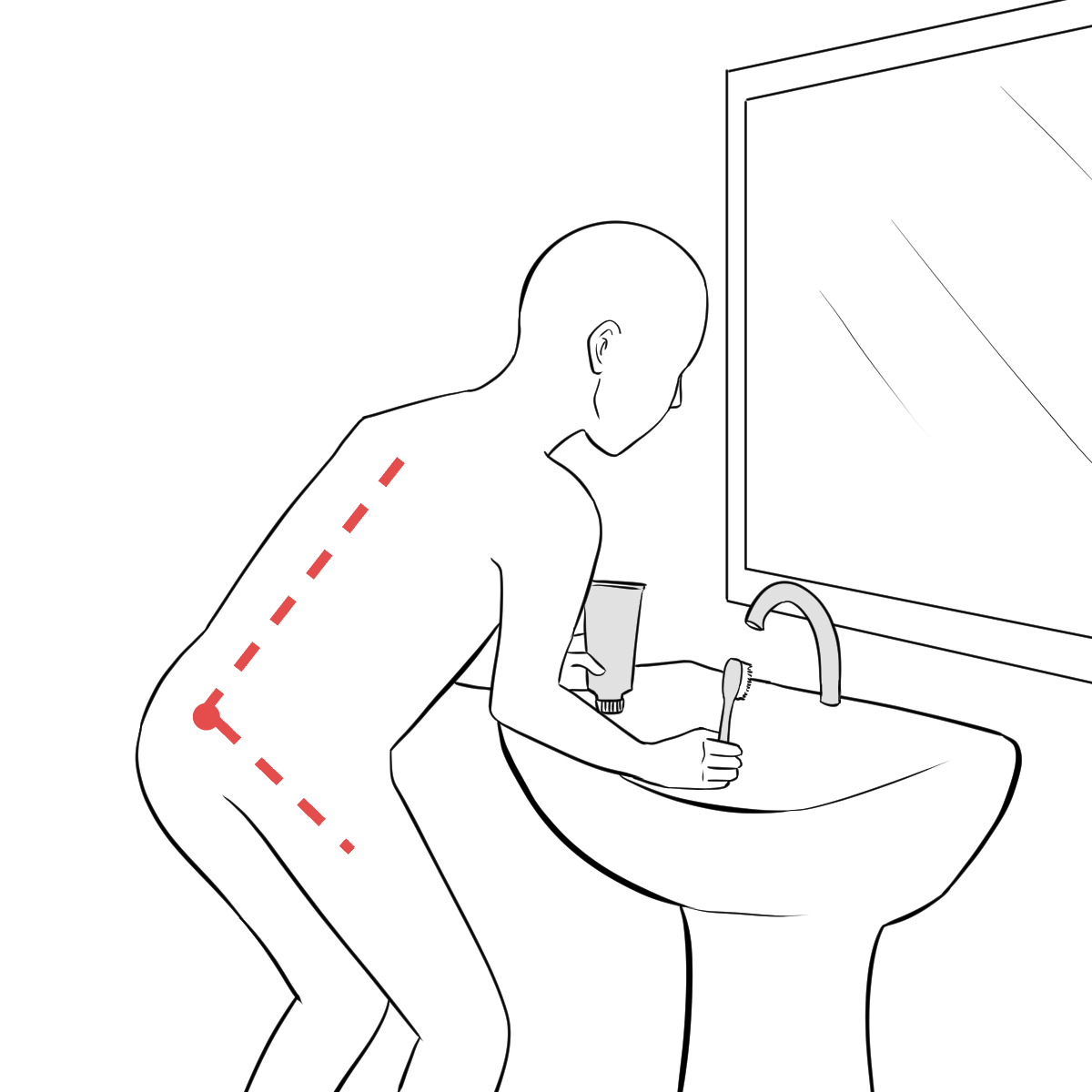

Supplement: Supplementary file 1 [file healthcare-12-02274-s001.zip › healthcare-3279036-supplementary/healthcare20241108_Postures/4-1_TIFF.tif]

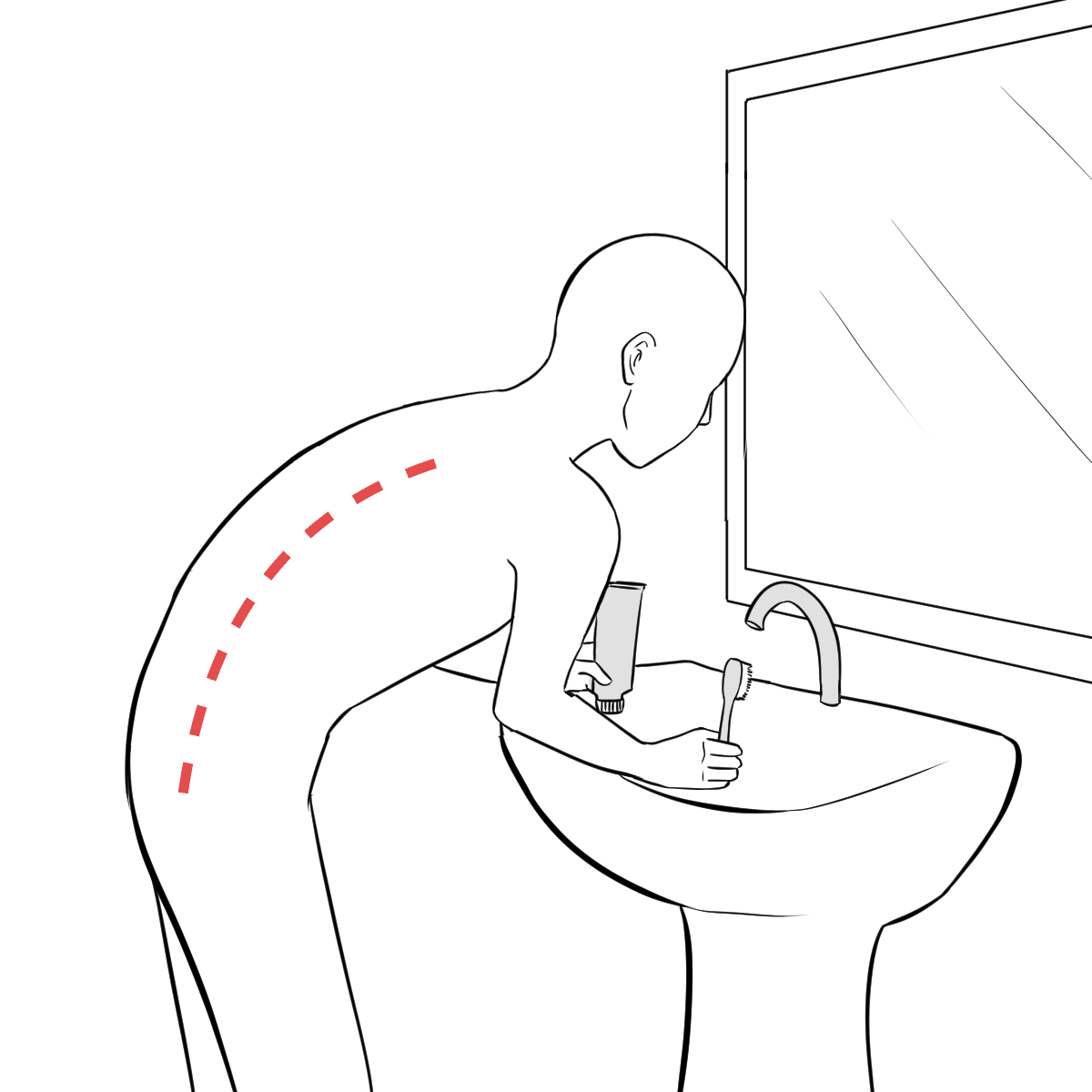

Supplement: Supplementary file 1 [file healthcare-12-02274-s001.zip › healthcare-3279036-supplementary/healthcare20241108_Postures/4-2.jpg]

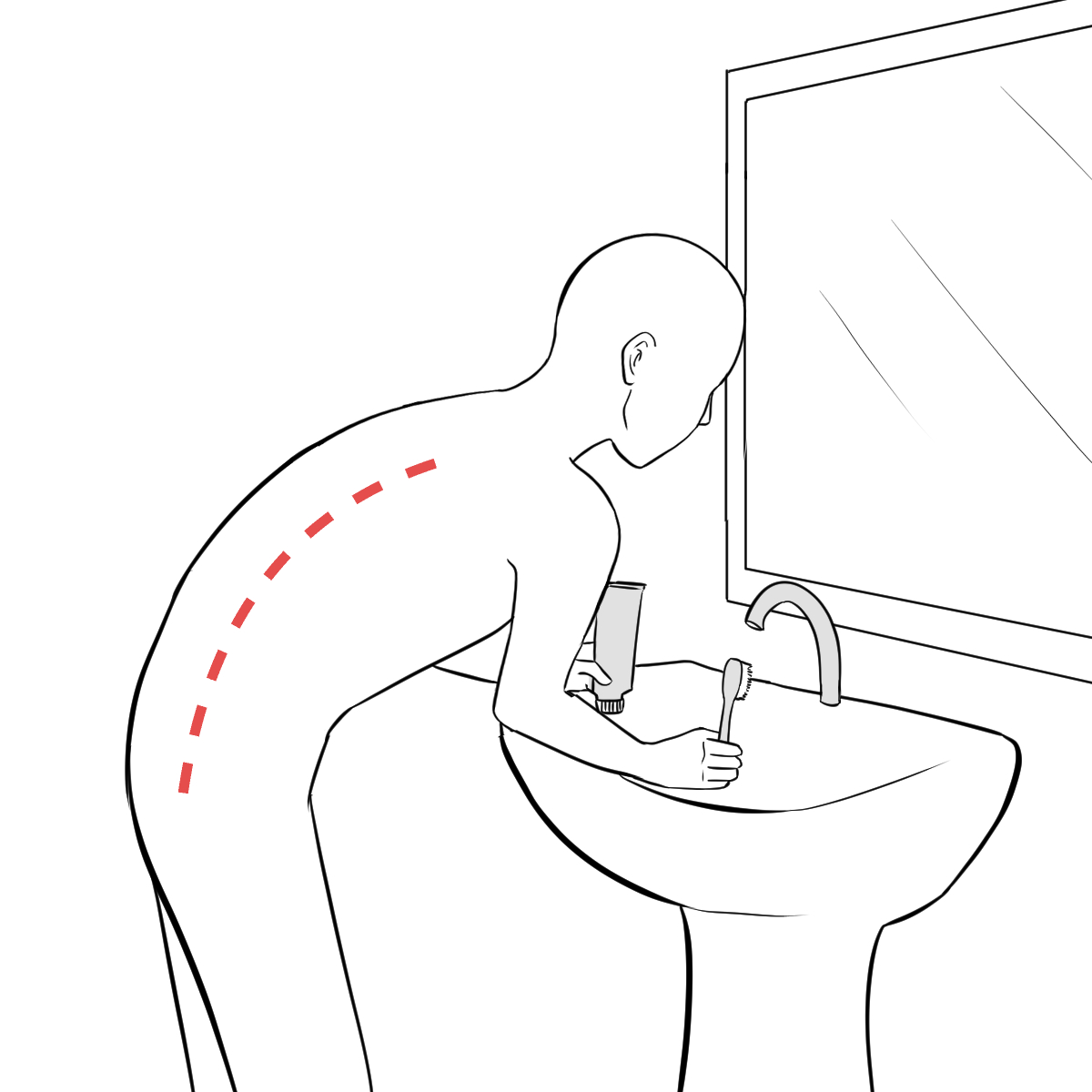

Supplement: Supplementary file 1 [file healthcare-12-02274-s001.zip › healthcare-3279036-supplementary/healthcare20241108_Postures/4-2_TIFF.tif]

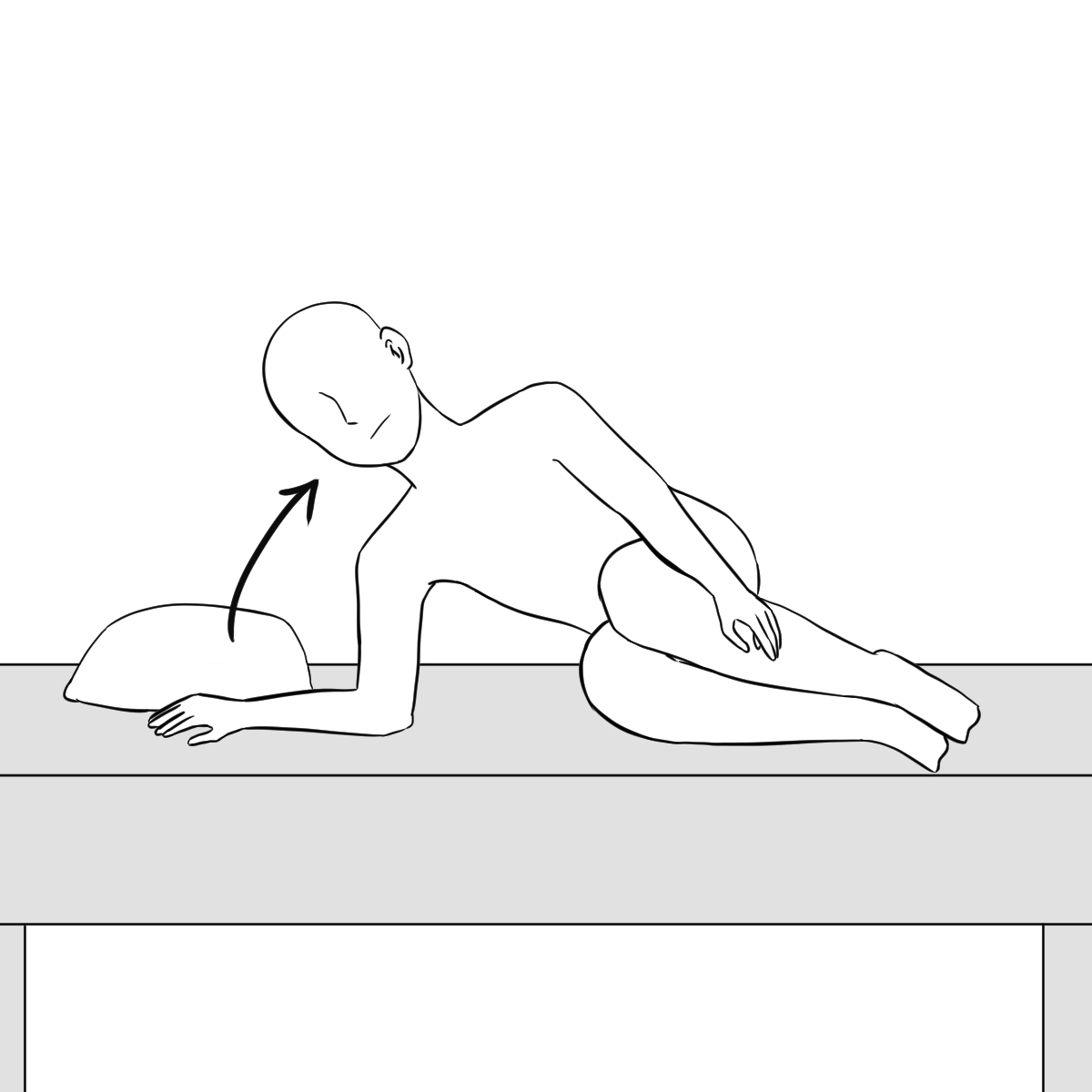

Supplement: Supplementary file 1 [file healthcare-12-02274-s001.zip › healthcare-3279036-supplementary/healthcare20241108_Postures/5-1.jpg]

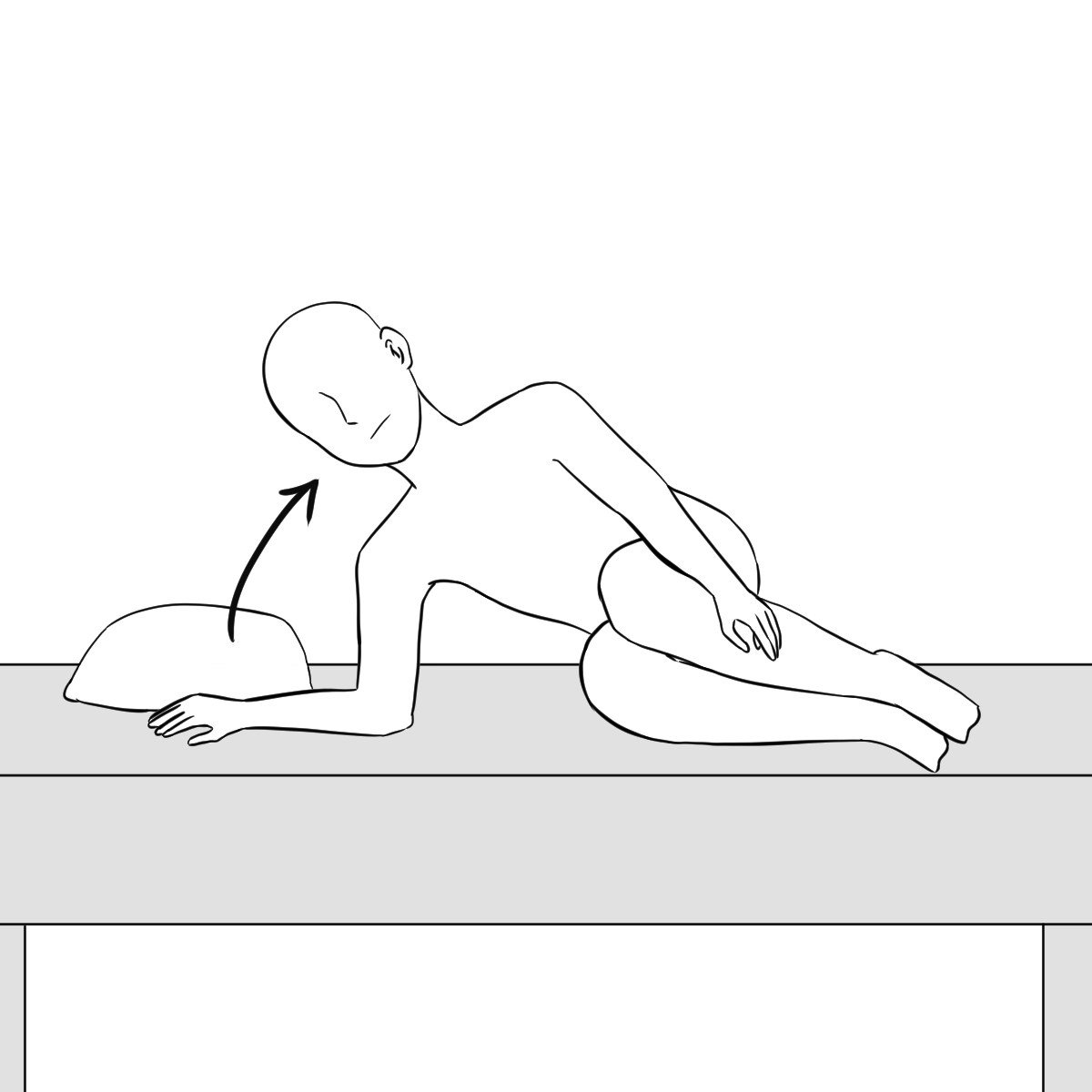

Supplement: Supplementary file 1 [file healthcare-12-02274-s001.zip › healthcare-3279036-supplementary/healthcare20241108_Postures/5-1_TIFF.tif]

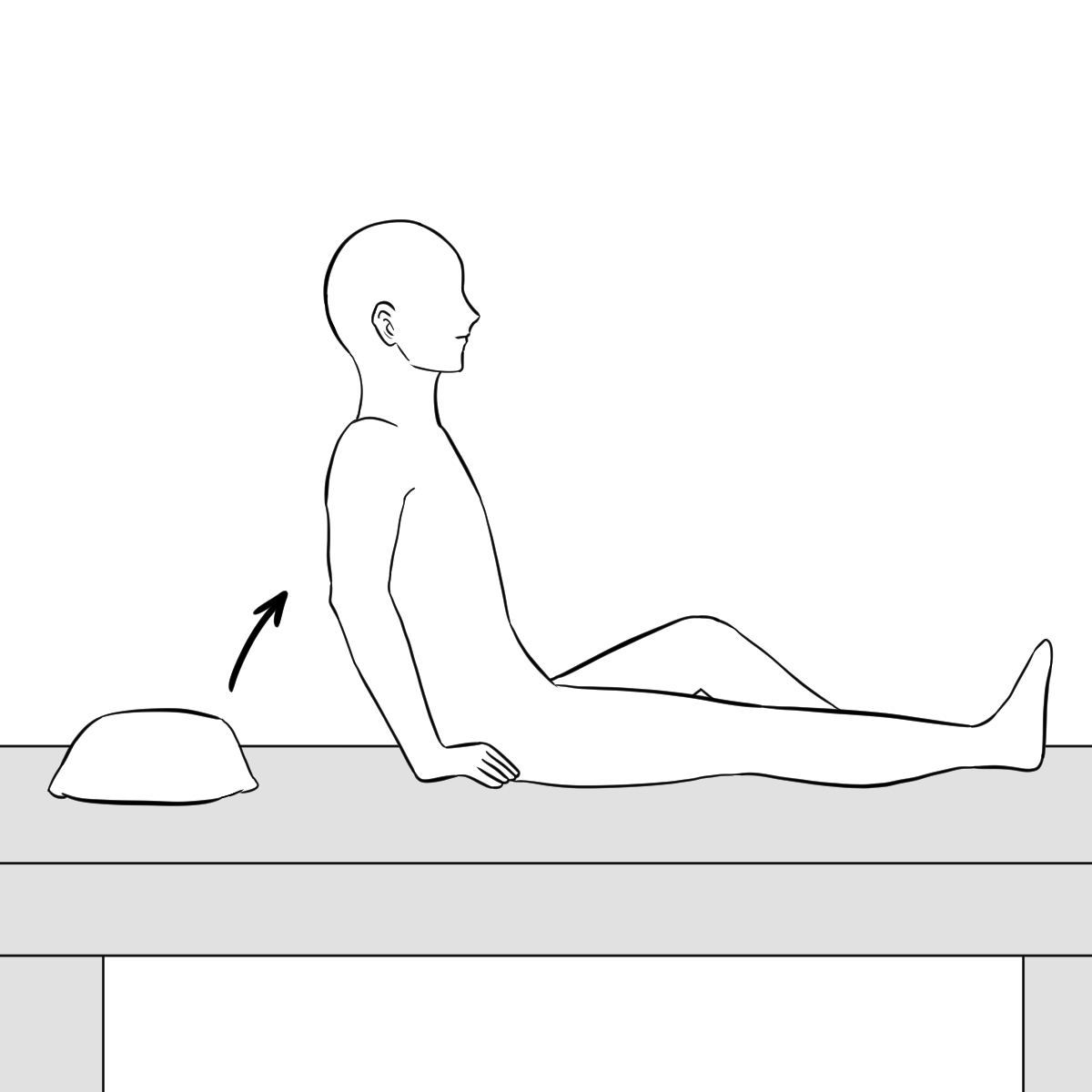

Supplement: Supplementary file 1 [file healthcare-12-02274-s001.zip › healthcare-3279036-supplementary/healthcare20241108_Postures/5-2.jpg]

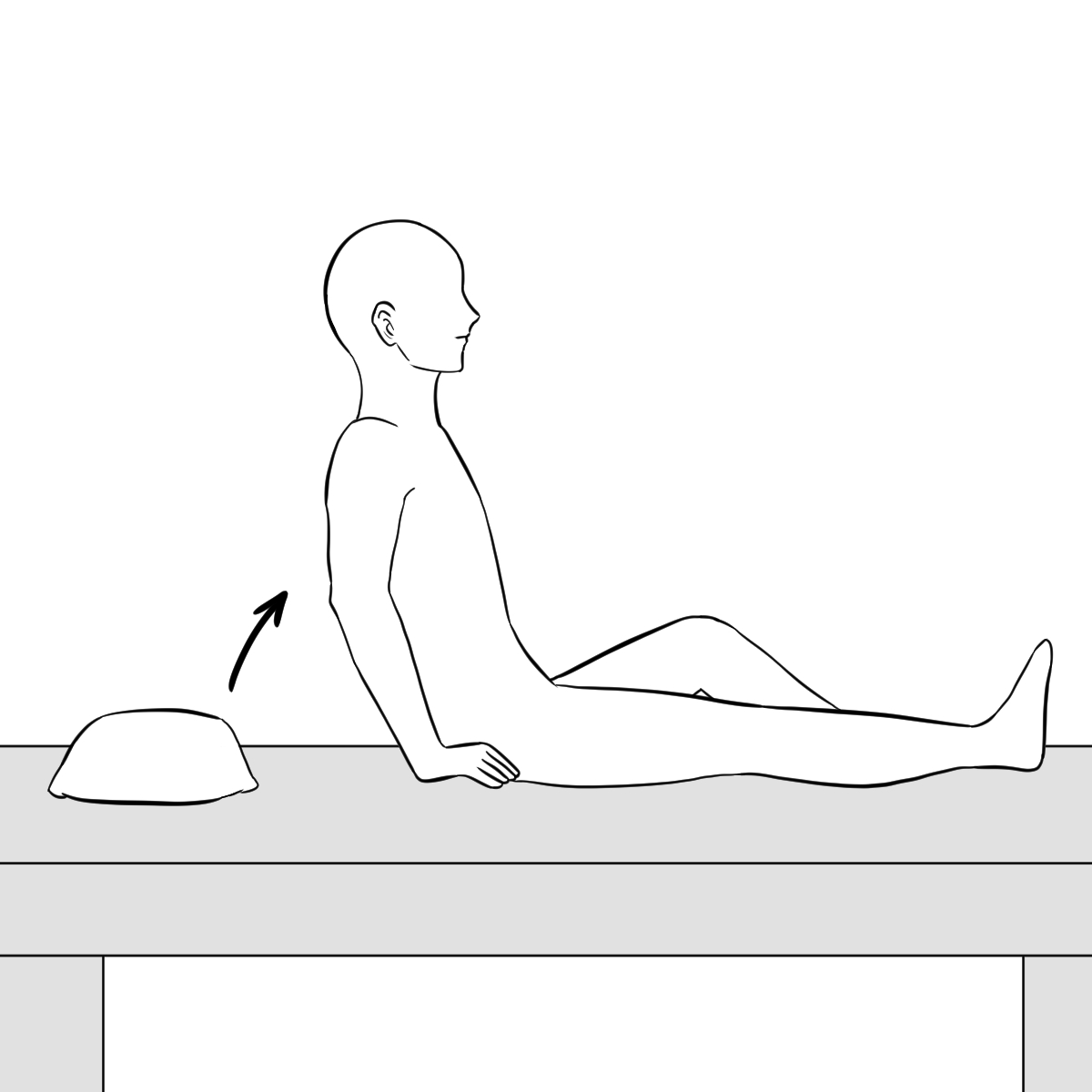

Supplement: Supplementary file 1 [file healthcare-12-02274-s001.zip › healthcare-3279036-supplementary/healthcare20241108_Postures/5-2_TIFF.tif]

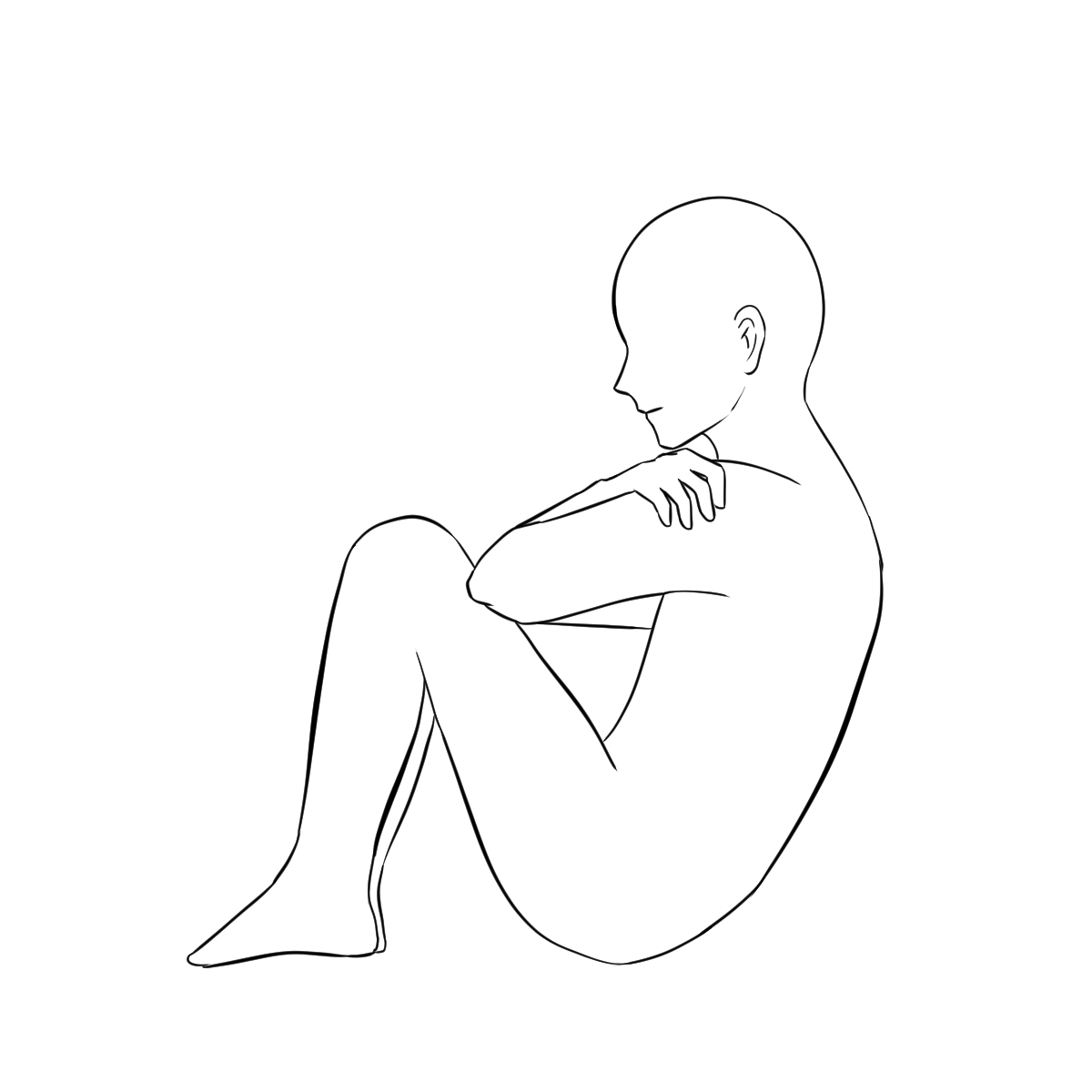

Supplement: Supplementary file 1 [file healthcare-12-02274-s001.zip › healthcare-3279036-supplementary/healthcare20241108_Postures/6-1.jpg]

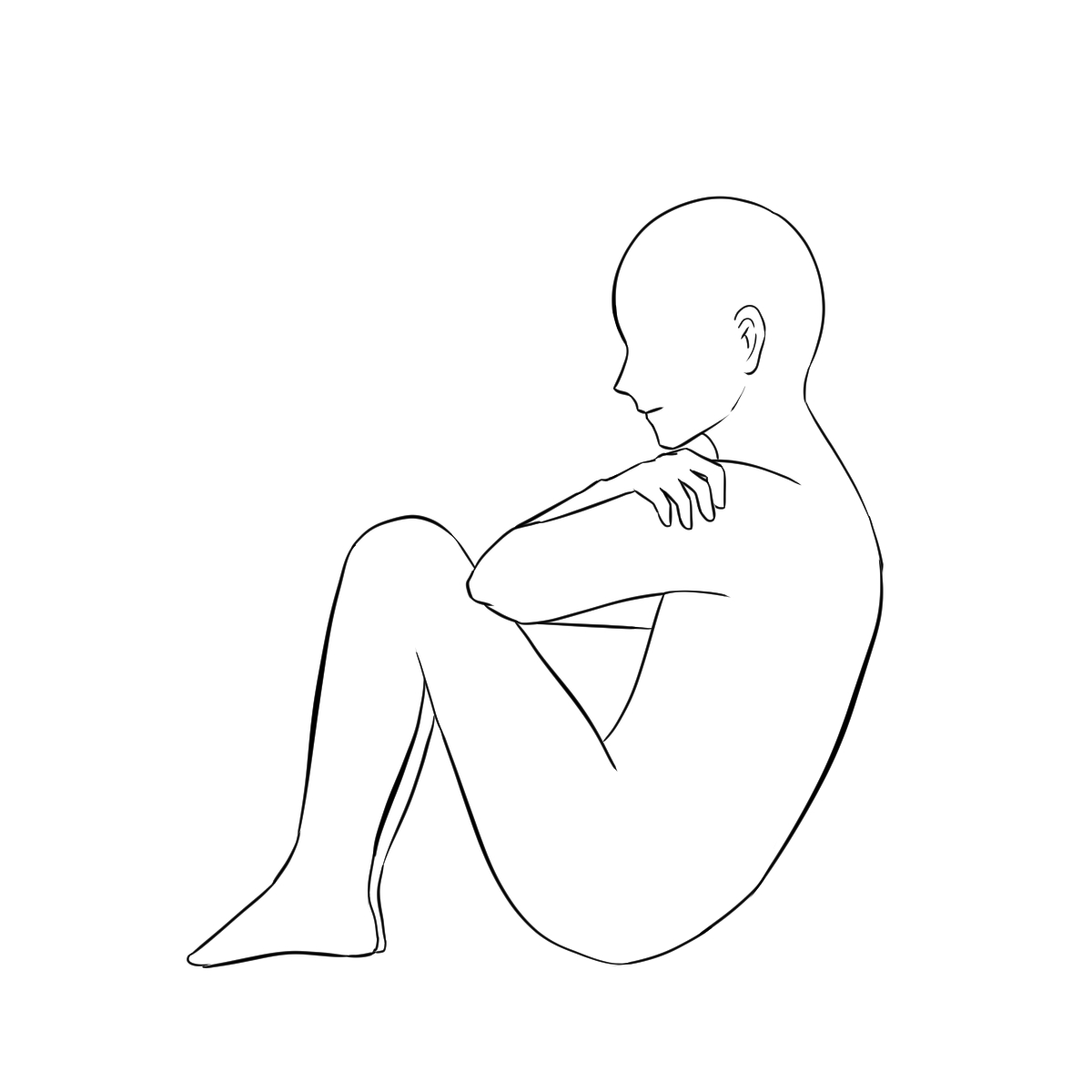

Supplement: Supplementary file 1 [file healthcare-12-02274-s001.zip › healthcare-3279036-supplementary/healthcare20241108_Postures/6-1_TIFF.tif]

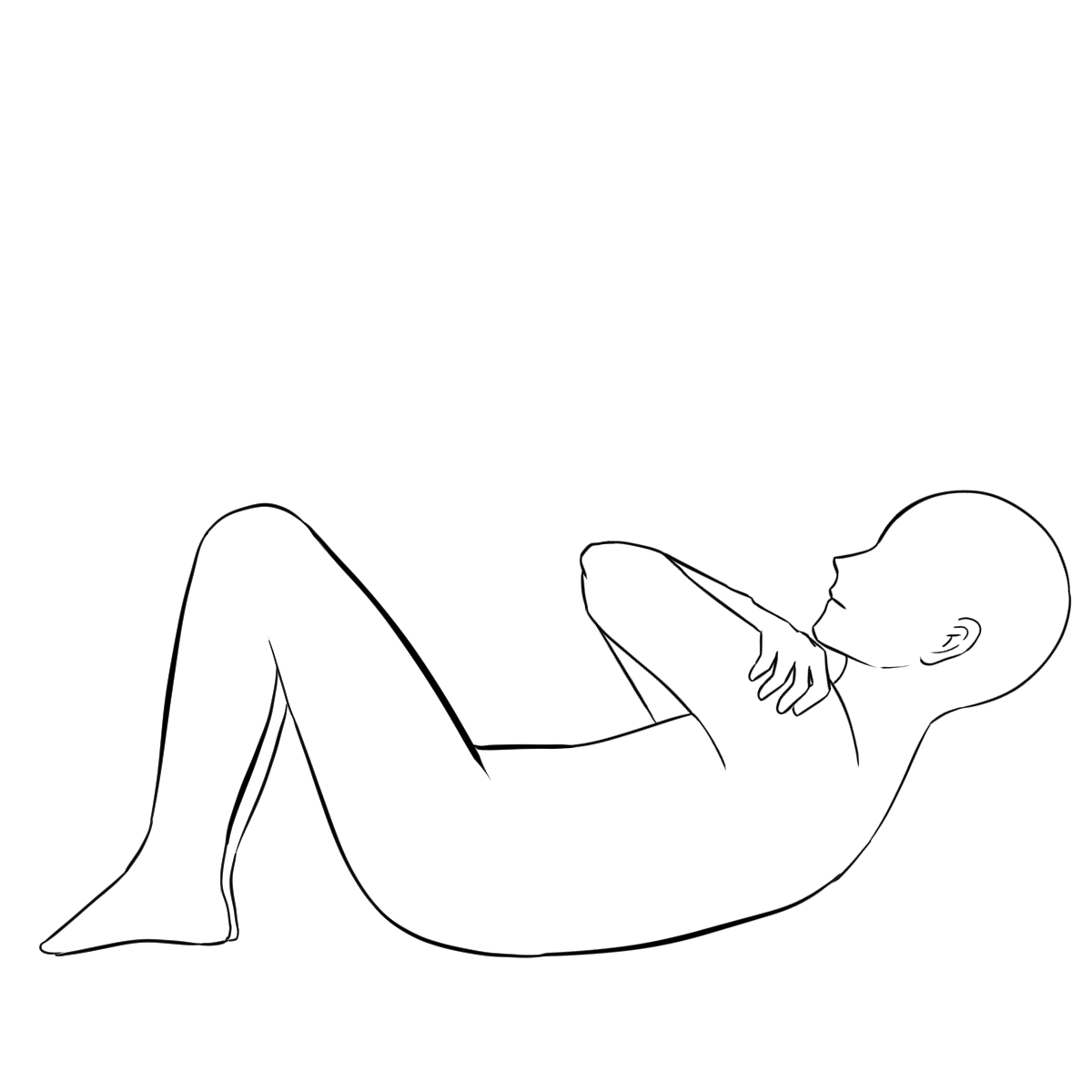

Supplement: Supplementary file 1 [file healthcare-12-02274-s001.zip › healthcare-3279036-supplementary/healthcare20241108_Postures/6-2.jpg]

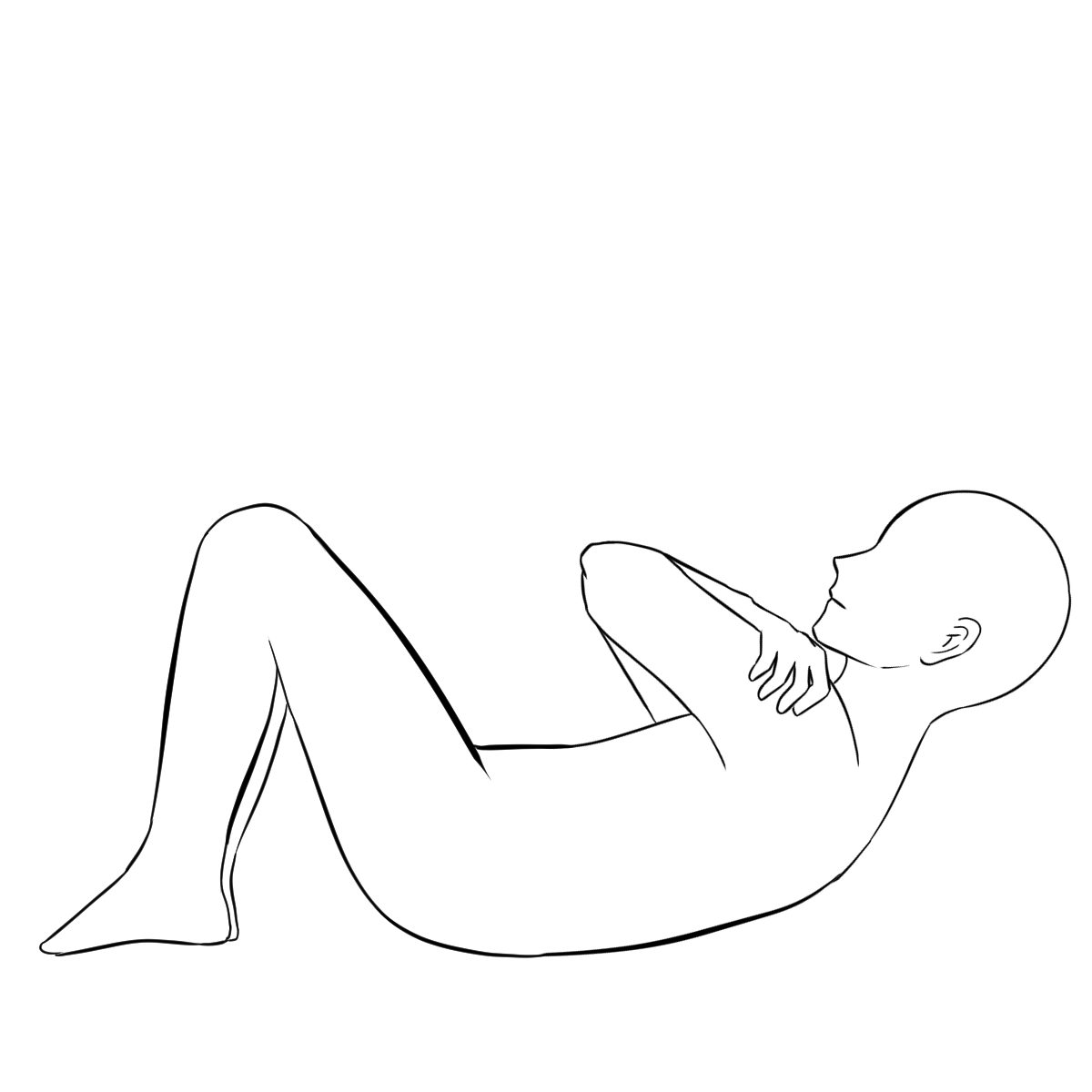

Supplement: Supplementary file 1 [file healthcare-12-02274-s001.zip › healthcare-3279036-supplementary/healthcare20241108_Postures/6-2_TIFF.tif]
